# A Systematic Review of Phytochemistry, Pharmacology and Pharmacokinetics on *Astragali* Radix: Implications for *Astragali* Radix as a Personalized Medicine

**DOI:** 10.3390/ijms20061463

**Published:** 2019-03-22

**Authors:** Zhenzhen Guo, Yanmei Lou, Muyan Kong, Qing Luo, Zhongqiu Liu, Jinjun Wu

**Affiliations:** 1 Joint Laboratory for Translational Cancer Research of Chinese Medicine of the Ministry of Education of the People’s Republic of China, International Institute for Translational Chinese Medicine, Guangzhou University of Chinese Medicine, Guangzhou, Guangdong 510006, China; guozhenzhenlcy@163.com (Z.G.); louyanmei321@163.com (Y.L.); kmy_1995n@163.com (M.K.); lqing1105@163.com (Q.L.); 2 State Key Laboratory of Quality Research in Chinese Medicine, Macau University of Science and Technology, Macau (SAR) 999078, China

**Keywords:** *Astragali* radix, phytochemistry, pharmacology, pharmacokinetics, personalized medicine

## Abstract

*Astragali* radix (AR) is one of the most widely used traditional Chinese herbal medicines. Modern pharmacological studies and clinical practices indicate that AR possesses various biological functions, including potent immunomodulation, antioxidant, anti-inflammation and antitumor activities. To date, more than 200 chemical constituents have been isolated and identified from AR. Among them, isoflavonoids, saponins and polysaccharides are the three main types of beneficial compounds responsible for its pharmacological activities and therapeutic efficacy. After ingestion of AR, the metabolism and biotransformation of the bioactive compounds were extensive in vivo. The isoflavonoids and saponins and their metabolites are the major type of constituents absorbed in plasma. The bioavailability barrier (BB), which is mainly composed of efflux transporters and conjugating enzymes, is expected to have a significant impact on the bioavailability of AR. This review summarizes studies on the phytochemistry, pharmacology and pharmacokinetics on AR. Additionally, the use of AR as a personalized medicine based on the BB is also discussed, which may provide beneficial information to achieve a better and more accurate therapeutic response of AR in clinical practice.

## 1. Introduction

*Astragali* radix (AR), also well-known as Huangqi in China, is one of the most popular herbal medicines that has been widely applied for over 2000 years in Chinese clinics. It is derived from the roots of *Astragalus membranaceus* (Fisch.) Bge. or *A. membranaceus* (Fisch.) Bge. var. *mongholicus* (Bge.) Hsiao. As a highly potent herb widely used to treat various diseases, AR has played an indispensable role in healthcare throughout Chinese history [1,2]. Traditionally, AR is used for the treatment of anemia, weakness, fever, wounds, chronic fatigue, multiple allergies, loss of appetite, uterine bleeding and uterine prolapse [3,4,5]. For these reasons, AR is used as an essential ingredient included in over 200 Chinese herb formulas [6]. Furthermore, AR is popularly consumed as a health food additive in some food to reinforce vital energy and body immunity [7]. Modern pharmacological studies and clinical practices indicate that AR possesses various biological functions, such as immunomodulation, antioxidant, anti-inflammation and antitumor activities [8]. Now, AR is typically prepared as a water extract either alone or together with other drugs to treat cardiovascular diseases, diabetes mellitus, cancer, respiratory diseases and nervous system diseases and so forth [9,10,11,12,13].

AR has been shown to contain isoflavonoids, triterpene saponins, polysaccharides and some trace elements [14]. To date, more than 200 compounds, including isoflavonoids, saponins, polysaccharides and amino acids, have been isolated and identified from AR and various biological activities of the compounds have been reported [15]. Among them, isoflavonoids, saponins and polysaccharides are the three main types of beneficial compounds responsible for the pharmacological activities and therapeutic efficacy of AR. The major isoflavonoids from AR, such as formononetin, ononin and calycosin and its glycoside, can strengthen the immune system, boost energy and promote health activities [16]. Astragaloside IV (AS-IV), a vital saponin compound from AR, possesses effective pharmacological activities and can be used as a marker compound for the quality assessment of AR [17]. In addition, more than 30 kinds of *Astragalus* polysaccharides (APS) have been identified [17]. What is more, some amino acids, vitamins and trace elements are also isolated and identified from AR [5,18,19]. 

Because of the extensive use of AR in clinical practice and its effective pharmacological functions, a large number of studies on the pharmacokinetic characteristics of AR and its bioactive compounds have been performed [20,21,22]. These studies clearly provide an insight into the pharmacokinetic process of the bioactive compounds in AR in vivo, which may help provide beneficial information for the safe and effective application of AR. From these studies, it is well-recognized that the metabolism and biotransformation of the bioactive compounds were extensive in vivo [20,23]. The isoflavonoids and saponins and their metabolites are the major type of constituents absorbed in plasma [20,23]. The bioavailability barrier (BB) in the body, which mainly consist of drug-metabolizing enzymes (DMEs) and efflux transporters (ETs), is expected to have a significant impact on the pharmacokinetic characteristics of AR [24,25,26]. In this paper, we give an overview and a critical assessment of the published data concerning the phytochemistry, pharmacology and pharmacokinetic studies on AR. Finally, the use of AR as a personalized medicine based on the BB is also discussed. 

## 2. Chemical Composition 

To date, more than 200 compounds have been isolated and identified from AR, including saponins, flavonoids, polysaccharides, amino acids, trace elements and other compounds [27]. Some of the main parts of compounds are displayed in Table 1. It has been confirmed that isoflavonoids, triterpene saponins and polysaccharides are the main bioactive compounds responsible for the various pharmacological properties of AR [28]. Knowing the different bioactive compounds of AR will provide beneficial information for better understanding of the different biological functions of AR. 

### 2.1. Astragalus Triterpene Saponins

AR abounds with saponins and the content of *Astragalus* total triterpene saponins in AR ranges from 0.5 mg/g to 3.5 mg/g [28]. With the development of separation, extraction and structure identification technologies, approximate 170 kinds of triterpenoid saponins have so far been isolated from AR [27]. As early as 1997, astrasieversianin II and astragaloside I, two cycloartane triterpene glycosides, were first isolated and identified in AR with spectroscopic analysis, chemical degradation and nuclear magnetic resonance analysis [32]. Then, eight saponins from AR were isolated. Based on spectral data, their chemical structures were further established as astrasieversianins II and X, astragalosides I, II, IV and VI and cyclocanthosides E and G [34]. Subsequently, three new cycloartane-type triterpene saponins, brachyosides A, B and C and one new glycoside, cyclocephaloside II, were isolated together with five known saponins, including astragalosides I, II and IV, cyclocanthoside E and cycloastragenol [45]. With the advance of technology and the importance attached to AR, more and more *Astragalus* saponins have been isolated and identified. Four new saponins were isolated and two of them were shown to stimulate the proliferation of mouse splenocytes without significant cytotoxic [38]. Then, four cycloartane- (hareftosides A–D) and oleanane-type triterpenoids (hareftoside E) were first identified and isolated from AR by the extensive use of 1D- and 2D-NMR experiments along with ESI-MS and HR-MS analyses [46]. Among the various *Astragalus* triterpene saponins, astragaloside I–VIII, acetyl astragaloside, isoastragaloside I–IV and soyasaponins are the major four types, which accounts for about 80% of all the saponins [28]. The studies on astragaloside IV (AS-IV) are more and more increasingly popular. A simple and fast quantification method for AS-IV has been established with ultra-performance liquid chromatography (UPLC-QDA) [47]. In addition, it has been confirmed that AS-IV is the main bioactive constituent among the *Astragalus* saponins, which has been reported to exert extensive pharmacological actions in various diseases [36]. Modern pharmacological experiments have indicated that AS-IV possesses cardiomyocyte protective, anti-aging, neuroprotective and antioxidative properties [48,49,50]. More importantly, AS-IV has been used as one of the vital markers for quality control of AR in Chinese Pharmacopoeia. 

### 2.2. Astragalus Flavonoids

It is well known that flavonoids are also one of the main types of beneficial compounds responsible for the biological activities of AR. It was determined that the content of *Astragalus* flavonoids in AR ranges from 0.5 mg/g to 3.0 mg/g [51]. So far, over 60 kinds of flavonoids compounds have been isolated, mainly including flavonoids, isoflavones, isoflavanes, rosetanes, flavonols, isoflavonols and dihydroisoflavones [27]. In 2000, UPLC-electrospray ionization mass spectrometry was applied to analyze the flavonoids of AR. Eight flavonoids were identified as calycosin-7-*O*-β-d-glucoside (CG), calycosin-7-*O*-β-d-glucoside-6″-*O*-malonate, ononin, (6aR,11aR)-3-hydroxy-9,10-dimethoxypterocarpan-3-*O*-β-d-glucoside, calycosin, (3R)-7,2′-dihydroxy-3′,4′-dimethoxyisoflavan-7-*O*-β-d-glucoside, formononetin-7-*O*-β-d-glucoside-6″-*O*-malonate and formononetin [52]. Among them, CG has been proved to exhibits several pharmacological activities, such as anti-inflammatory, antioxidative and neuroprotective activities [53,54]. More importantly, CG has been used as another chemical indicator in the quality control of AR in Chinese Pharmacopoeia (2015). In addition, formononetin and calycosin, another two important isoflavonoid components present in AR, have also been extensively studied because of their effective and various pharmacological functions [55,56]. What is more, three special *Astragalus* flavonoids, including pendulone, isoliquiritigenin and sulfuretin, have also been isolated from AR [27]. 

### 2.3. Astragalus Polysaccharides (APS)

It is relatively difficult to isolate and characterize the individual APS because of their macromolecules with complicated chemical structures. To date, over 30 APS have been isolated and are mainly classified into dextran and heteropolysaccharides [27]. From a study in 2004, the APS was extracted from AR with the method of water extraction-alcohol precipitation. The molecular weight of the extract was about 3500–1.58 × 10^6^ as determined by the gel filtration method. Sugar compositional analysis by TLC and gas chromatography showed that it consisted of Rha, Xyl, Glc, Gal, Man and Fru in the molar ratios of Rha: Xyl: Glc: Gal: Man: Fru = 4.9: 4.7: 8.3: 122.2: 2.2: 3.1 [42]. In another study, the alcohol-soluble polysaccharide (ASP) was also extracted from AR and their preliminary structural characteristics were investigated. The contents of total sugar, protein and uronic acid in ASP was 92.04%, 0.51% and 1.42%, respectively. Further analysis indicated that ASP (about 2.1 × 10^3^ Da) was a neutral polysaccharide composed of arabinose, galactose, glucose and mannose (molar ratio: 1.00:0.98:3.01:1.52) with pyranose ring and α-type glycosidic linkages [5]. Recently, a new cold-water-soluble polysaccharide was extracted from AR and was named as APS4. The average molecular weight of APS4 was approximately 1.5 × 10^6^ Da determined by high-performance gel-permeation chromatography (HPGPC) analysis. APS4 is consists of rhamnose, arabinose, xylose, mannose and galactose, in a molar ratio of 12.1:0.3:0.6:1.0:1.0:1.7 through Chromatography (GC) analysis. More experiments indicated that APS4 had the potential application for cancer treatment [43]. The authors further investigated the effects of different temperatures on structural characterization of polysaccharides from AR by specific rotation and scanning electron microscope (SEM) analysis [57]. In addition, a novel polysaccharide named as AERP was extracted from industrial AR-extracted waste residue, which was composed of two components coded as AERP1 and AERP2. Moreover, the structure of the two components was determined by HPLC-SEC-RID, HPLC-Ci8-UV, FT-IR and NMR analysis. AERP1 was an acidic component with a molecular weight of 2.01 × 10^6^ Da and AERP2 was a glucan with 2.11 × 10^3^ Da [39]. 

### 2.4. Other Constituents

In addition to the isoflavonoids, saponins and polysaccharides, AR also contains over 20 kinds of trace elements such as rubidium, manganese, copper, chromium, cobalt, scandium, selenium, cesium, iron, molybdenum and zinc [8]. In addition, AR also contains 20 types of amino acids, including canavanine, arginine, aspartic acid, asparagine, proline and alanine [42]. What is more, other compounds, such as ferulic acid, palmitic acid, coumarin, folic acid, bitter elements, choline, linolenic acid, vanillic acid, isoferulic acid, hydroxy phenyl acrylic acid, caffeic acid, daucosterol, lupeol, betaine, linoleic acid, green acid, palm acid and 13-sitosterol have also been identified in AR. 

## 3. Pharmacological Activities

As a highly potent herb widely used to treat various diseases, AR has played an indispensable role in healthcare throughout Chinese history [1,2]. Traditionally, AR is used for the treatment of anemia, weakness, fever, wounds, chronic fatigue, multiple allergies, loss of appetite, uterine bleeding and uterine prolapse [3,4,5]. Modern pharmacological studies and clinical practices indicate that AR and its main active ingredients possess various biological properties and are widely used to treat cardiovascular diseases, diabetes mellitus, cancer, respiratory diseases and nervous system diseases [8].

### 3.1. Effects of AR and Its Main Components on Cardiovascular Diseases

**Figure 1:**
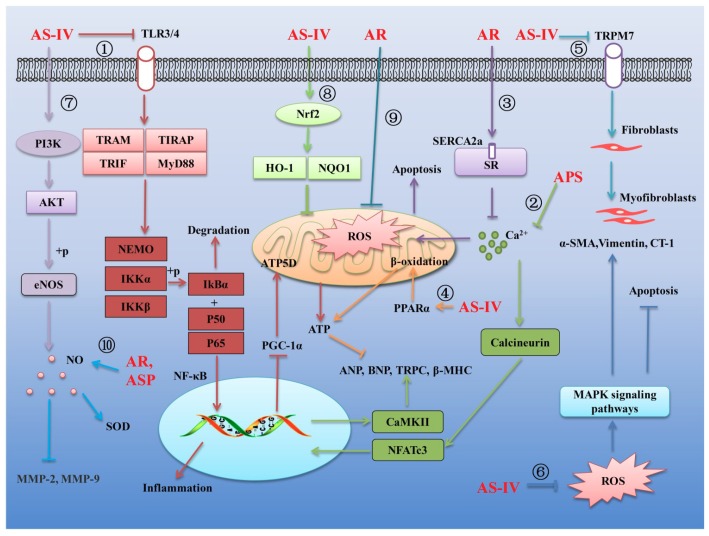
Several mechanisms linking AR and its main components to the treatment of cardiovascular diseases. Arrows and bar-headed lines represent signaling activation and inhibition, respectively. The different signaling pathways regulated by AR and its components are numbered ① to ⑩.

An increasing number of experiments have confirmed that AR can effectively inhibit cardiovascular diseases such as myocardial ischemia-reperfusion injury [9], myocardial hypertrophy [58], vascular endothelial dysfunction [59], coronary heart disease [60], atherosclerosis [61], cardiac fibrosis [62] and viral myocarditis [63]. The main mechanisms of the treatment of cardiovascular diseases by AR and its main components are summarized in Figure 1.

Myocardial hypertrophy is usually considered as a compensatory response to pathological conditions [64]. Long-term hypertrophy can lead to myocardial inflammation, myocardial metabolic disorders, abnormal energy production of myocardial cells, myocardial ischemia and hypoxia, heart failure and sudden death [64]. It is well known that nuclear factor κB (NF-κB) is a family of transcription factors, which have an effect on regulation of inflammatory response [65,66,67,68]. Growing evidence suggests that activation of Toll-like receptor 4 (TLR4) receptor can activate its downstream NF-κB signaling pathway, thereby promoting the production of inflammatory factors in damaged myocardium cells. It was reported that AS-IV could attenuate inflammatory cytokines by inhibiting TLR4/NF-κB signaling pathway in isoproterenol-induced myocardial hypertrophy in rats [69] (Figure 1①). On the other hand, dysfunction of cardiac energy biosynthesis contributes to the hypertrophy and NF-κB/Peroxisome Proliferator-activated Receptor-γ Coactivator 1α (PGC-1α) signaling gets involved in the dysfunction. The results from another study showed that AS-IV could protect against isoproterenol-induced cardiac hypertrophy through regulating NF-κB/PGC-1α signaling mediated energy biosynthesis [70] (Figure 1①). Moreover, it is known that calcium homeostasis plays an important role in the progression of myocardial hypertrophy. When cardiac hypertrophy occurs, overload calcium can activate its downstream calcineurin and promote the nuclear factor of activated T cells cytoplasmic 3 (NFATc3) transcription factor into the nucleus, then activate calmodulin II kinase (CaMKII) and promote the expression of cardiac hypertrophy indicators. It was found that APS could alleviate the augment of intracellular free calcium during cardiac hypertrophy induced by isoproterenol. The upregulated expression of calcineurin, translocation of nuclear factor of activated T cells, NFATc3 into nucleus and activation of CaMKII (reflected by p-CaMKII) were suppressed by the application of APS. These results revealed that APS can exert its anti-hypertrophic action via inhibiting Ca^2+^-mediated calcineurin/NFATc3 and CaMKII signaling cascades in isoproterenol-induced cardiac hypertrophy rats [71] (Figure 1②).

Sarcoplasmic reticulum (SR) plays an important role in the regulation of intracellular calcium. Sarcoplasmic reticulum Ca^2+^-ATPase (SERCA2a) in SR is responsible for the re-uptake of sarcoplasmic calcium. Numerous studies have shown that the expression of SERCA2a was markedly decreased in injured myocardium. It was reported that AR can promote the expression of SERCA2a, promote the re-uptake of calcium in SR, inhibit overload calcium, thereby inhibiting oxidative stress-induced cell apoptosis and cytoskeleton damage in adriamycin-injured rat hearts [72] (Figure 1③). In another study on the effects of AS-IV on heart failure, the authors found that AS-IV can activate peroxisome proliferator-activated receptor alpha (PPARα) to stimulate fatty acid β-oxidation and increase cardiac energy production, improving mitochondrial function and the efficiency of SERCA in heart failure in pressure overload-induced HF mice and isolated hypertrophic myocardial cells [73] (Figure 1④).

Cardiac fibroblasts represent 65–70% of total cells in the heart. The pathological characteristics of cardiac fibrosis are an infinite proliferation of fibroblasts and excessive deposition of extracellular matrix (ECM) proteins in the myocardium [74]. In a study of inhibiting hypoxia-induced myocardial fibrosis by AS-IV, it was observed that AS-IV could inhibit transient receptor potential cation channel, subfamily M, member 7 (TRPM7), decrease the intracellular calcium, decrease the α-smooth muscle actin (α-SMA, a marker of fibroblast differentiation), inhibit fibroblasts differentiation into fibroblasts and inhibit the expression of ECM and collagen I (a marker of cardiac fibroblasts) in vivo and in vitro [74] (Figure 1⑤). Oxidative stress is well recognized as a common feature of cardiac fibrosis. In damaged myocardium, excessive reactive oxygen species (ROS) can activate mitogen-activated protein kinases (MAPKs) signaling pathway and then promote the phosphorylation of the three profibrotic MAPKs, namely extracellular signal-regulated kinase (ERK), p38MAPK and c-Jun N-terminal kinase (JNK). Activation of the MAPKs signaling pathway can further regulate the expression of nuclear transcription factors on fibroblast-promoting factors and ECM proteins. AS-IV has been demonstrated to possess remarkable antifibrotic effects via its antioxidative activity. It was reported that AS-IV may inhibit isoproterenol-induced cardiac fibrosis by suppressing ROS-mediated MAPK activation [75]. Moreover, excessive ROS can promote the production of cardiotrophin-1 (CT-1), which can significantly promote cardiac fibrosis. AS-IV was also able to effectively inhibit isoproterenol-induced cardiac fibroblast proliferation and collagen production through negative regulation of ROS-mediated CT-1 upregulation [62] (Figure 1⑥).

Coronary heart disease (CHD) is another major cardiovascular disease, which remains a serious public health burden [76]. The vasoreactivity of the endothelial-dependent coronary artery is a key indicator of vascular function. Endothelial dysfunction is characterized by decreased nitric oxide (NO) bioavailability [77]. It is well known that NO, which is mainly produced by endothelial NO synthase (eNOS), is a major endothelium-derived mediator controlling vascular dilation and plays an important role in physiological and pathological endothelial cells function [78]. It was reported that AS-IV exerted a vasodilator effect on the aortic rings and increased the NO content by enhancing the eNOS release via the phosphatidylinositol3-kinase (PI3K)/protein kinase B (Akt)/eNOS signaling pathway [76] (Figure 1⑦). AS-IV was also was found to activate nuclear factor-erythroid 2-related factor 2 (Nrf2) signaling pathway to promote the expression of antioxidant factors oxygenase-1 (HO-1) and NADPH (NQO1), thereby scavenging ROS levels and inhibiting the oxidative stress injury in lipopolysaccharide (LPS)-induced vascular endothelial cell injury mice [79] (Figure 1⑧). In another study, the effects of the AR extracts and their main compounds on mitochondrial bioenergetics were evaluated. The results showed that AR water extract inhibited the ROS production and possessed the strongest antioxidant activity and protective effects in cultured cardiomyocyte H9C2 cells exposed to oxidative stress. This protection was proposed to be mediated by increasing the spare respiratory capacity and mitochondrial ATP production in the stressed cells [80] (Figure 1⑨). Moreover, metalloproteinases are closely related to hyperhomocysteinemia induced cardiovascular disease. It was observed that total extract of AR and Astragalus saponins (ASP) could increase the production of NO in abnormal aorta, increase the activity of superoxide dismutase (SOD) and decrease the concentration of metalloproteinases MMP-2 and MMP-9, thereby improving vascular endothelial dysfunction in hyperhomocysteinemia induced cardiovascular disease rats [81] (Figure 1⑩).

### 3.2. Effects of AR and its Main Components on Diabetes Mellitus

**Figure 2:**
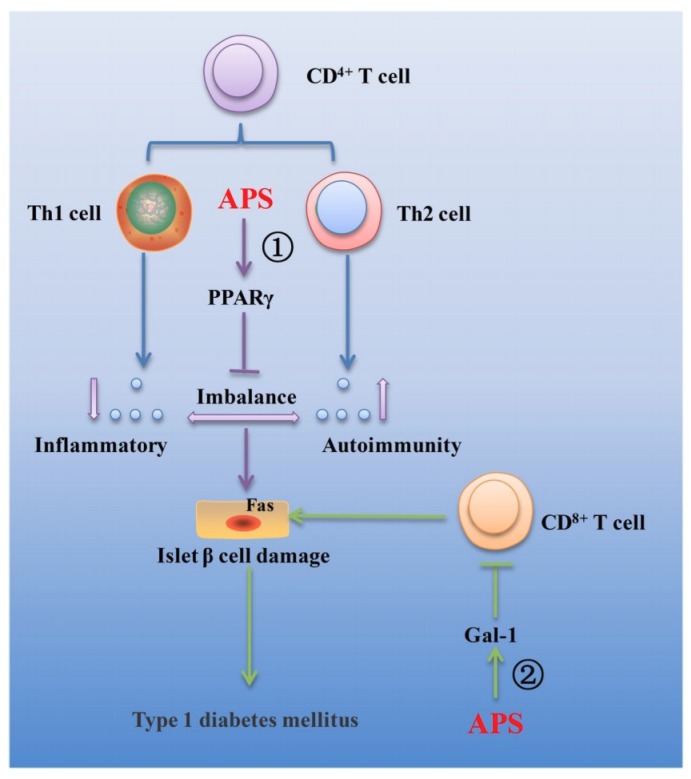
Several mechanisms linking AR and its main components to the treatment of type 1 diabetes mellitus. Arrows and bar-headed lines represent signaling activation and inhibition, respectively. The different signaling pathways regulated by AR and its components are numbered ① to ②.

**Figure 3:**
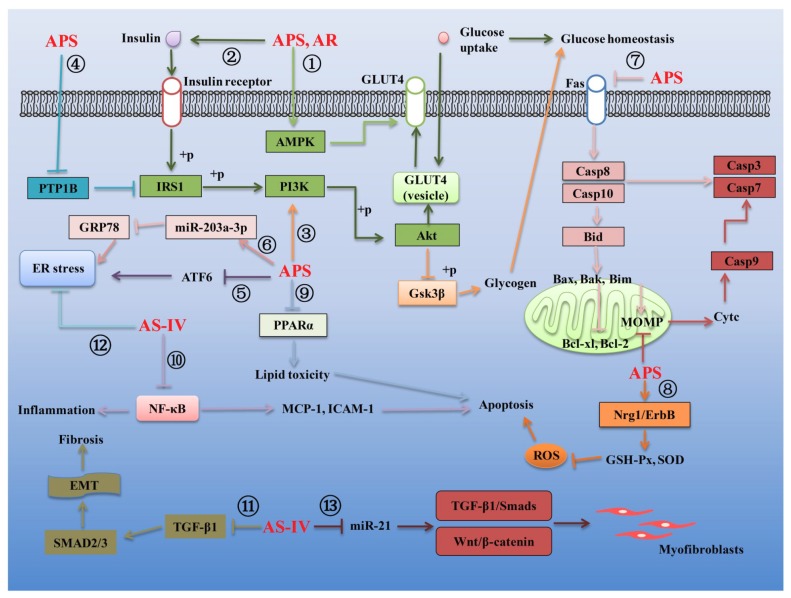
Several mechanisms linking AR and its main components to the treatment of type 2 diabetes mellitus. Arrows and bar-headed lines represent signaling activation and inhibition, respectively. The different signaling pathways regulated by AR and its components are numbered ① to ⑬.

Type 1 diabetes mellitus (T1DM) and type 2 diabetes mellitus (T2DM) are widely prevalent metabolic diseases with differing pathologies. T1DM manifests due to autoimmune destruction of the pancreatic beta cells, resulting in a diminished secretion of insulin. T2DM originates from a state of insulin resistance, resulting in hyperglycemia and reduction in beta cell mass. Both diseases can cause severe health consequences [82]. Compelling studies have demonstrated that AR and its main components possess effective biological function against T1DM and T2DM. Several mechanisms linking AR and its main components to the treatment of the T1DM and T2DM are summarized in Figure 2 and Figure 3, respectively.

#### 3.2.1. Effects of AR and Its Main Components on T1DM

T1DM is an autoimmune disease characterized by the destruction of insulin-producing beta cells in the pancreas and the absolute insufficiency of insulin secretion [83]. T lymphocyte (T cell) mediated immune response plays an important role in the pathogenesis of T1DM. The main functional subgroups of T cell include CD^4 +^ T cells and CD^8 +^ T cells. Under normal physiological conditions, CD^4 +^ T cells and CD^8 +^ T cells maintain a dynamic balance of CD^4 +^/CD^8 +^ and keep a normal immune response in the body. Under the stimulation by antigens, the CD^4 +^ T cells differentiate into Th1 and Th2 cells. Those cytokines secreted by Th1 cells include IL-1, IL-2, IL-6, IL-12, TNF-α and INF-γ, which can mediate the cellular immunity and cause damage to islet beta cells. Those cytokines secreted by Th2 cells include IL-4, IL-5, IL-6, IL-10, IL-13 and TGF-β, which can mediate fluid immunity and protect the islet beta cells. Th1 and Th2 cytokines together play important roles in immune regulation [84]. In clinics, APS has been widely used as an immunosuppressive agent. Substantial evidence exists that APS could downregulate blood glucose level, upregulate serum insulin concentration, increased beta cell mass and decrease apoptotic beta cell percentage, resulting in the downregulation of Th1/Th2 cytokine ratio and upregulation of PPARγ gene expression in spleens of streptozotocin-induced T1DM mice [85,86,87]. In addition, it was also demonstrated the effects of APS on the prevention of T1DM by correcting the imbalance between the Th1/Th2 cytokines on non-obese diabetic (NOD) mice [88] (Figure 2①). Furthermore, it is known that galectin-1 (gal-1) is closely related to T cell activation and apoptosis. It was found that APS could up-regulate the expression of galectin-1 in muscle of streptozotocin-induced T1DM mice, resulting in the apoptosis of CD^8 +^ T cells, which may be an important mechanism by which APS protects β cells of the pancreatic islets from apoptosis induced by CD^8 +^ T cells in T1DM in vivo [89] (Figure 2②).

#### 3.2.2. Effects of AR and Its Main Components on T2DM

Substantial evidence exists that improving insulin tolerance is the main mechanism of AR and its active ingredients in the treatment of T2DM. Glucose transporter 4 (GLUT4), which is responsible for sugar transport, is related to insulin sensitivity in different tissues and organs. On the one hand, the expression of GLUT4 can be regulated by the AMP-activated protein kinase (AMPK). On the other hand, the binding of insulin and insulin receptor can induce the phosphorylation of insulin receptor substrate 1 (IRS1), then activate PI3K/Akt signaling pathway and finally induce the GLUT4 expression. It was demonstrated that APS could not only activate the AMPK signaling pathway to promote GLUT4 expression and glucose metabolism (Figure 3①) but could also activate the insulin signaling pathway to promote GLUT4 from intracellular vesicles to cell membranes, thereby enhancing insulin sensitivity in mouse 3T3-L1 preadipocytes [90,91,92] (Figure 3②). Similarly, it was also observed that the AR water extract could prevent the development of diabetes and improves renal function in the T2DM db/db mice and the regulation of the IRS1-PI3K-GLUT signaling pathway by AR water extract could significantly improve diabetic nephropathy [10] (Figure 3②). Furthermore, glycogen synthase kinase-3 (GSK-3β) is now recognized as a key component of a surprisingly large number of cellular processes and diseases. Dysregulation of GSK3-3β is linked to several prevalent pathological conditions, such as diabetes and/or insulin resistance [93]. It has been revealed that the activation of the PI3K/Akt signaling pathway can produce a inhibition on the GSK3 activity [94]. The evidence linking activation of APS on the PI3K/Akt signaling pathway led to the hypothesis that this compound may play a key role in regulating the glucose homeostasis through the PI3K/Akt/GSK-3β signaling pathway (Figure 3③). Evidence also exists that protein tyrosine phosphatase 1B (PTP1B) plays an important role in insulin resistance. PTP1B can inhibit the phosphorylation of IRS1 in insulin signaling pathway and then promote insulin resistance. It was reported that APS enables insulin-sensitizing and hypoglycemic activity at least in part by decreasing the elevated expression and activity of PTP1B in the skeletal muscles of streptozotocin-induced T2DM rats [95] (Figure 3④). What is more, endoplasmic reticulum (ER) is an organelle responsible for regulating the synthesis of membrane proteins and regulating secretory organs. Pathological stress state can disturb the homeostasis of ER, leading to unfolded and misfolded ER lumen and ER stress response. The cumulative results show that ER stress response is also one of the main characteristics of T2DM. Transcription activator 6 (ATF6) and PTP1B play important roles in regulating ER stress response. It was concluded that that APS can inhibit ER stress by inhibiting ATF6 activation and PTP1B expression in streptozotocin-induced T2DM rats [96,97] (Figure 3⑤).

The cumulative findings show that microRNAs also play a vital role in regulating the expression of insulin secretion-related proteins. MicroRNAs microarray combined with bioinformatics has been used to screen differentially expressed micro (mi)RNAs after treatment with APS in T2DM Goto Kakizaki (GK) rats. By bioinformatics analysis, the authors found 12 differentially expressed microRNAs. The findings further provided evidence that miRNA-203a-3p, the most prominent differentially expressed gene, may have a functional role in ER stress signaling in the liver of T2DM GK rats. In addition, APS attenuated insulin resistance in T2DM, likely through upregulating or maintaining the miR-203a-3p expression levels, decreasing its target GRP78 mRNA and protein expression levels and regulating the protein expression of the ER stress signaling pathway [98] (Figure 3⑥).

A growing number of studies suggested that AR and its active ingredients also possess effective curative effects on those diabetic complications, including diabetic cardiomyopathy, diabetic nephropathy, diabetic retinopathy and cognitive dysfunction. For example, diabetic cardiomyopathy is characterized by an imbalance between myocyte death and regeneration mediated by the progressive loss of cardiac stem and progenitor cells (CSPCs) by apoptosis and necrosis due to the activation of oxidative stress with diabetes. It was confirmed that APS could increase the CSPCs abundance by the inhibition of oxidative stress-mediated apoptosis in diabetic hearts of streptozotocin (STZ)-induced diabetic mice [99]. Substantial evidence exists that diabetes-induced cardiomyocyte apoptosis is mainly mediated by intrinsic mitochondrial-mediated apoptotic signaling pathway and extrinsic death receptor-mediated apoptotic signaling. The change of mitochondrial extracorporeal membrane permeability (MOMP) can promote the release of cytochrome C from mitochondria to the cytoplasm and then promote the expression of apoptotic-related proteins Caspase 9 and Caspase 3, which play a vital role in activating the intrinsic mitochondrial-mediated apoptotic signaling pathway. Inflammatory factors such as TNF-α can activate Fas death receptors and then promote the expression of pro-apoptotic proteins Caspase 8, Caspase10, Bid, Bax, Bak and Bim, while inhibiting the expression of anti-apoptotic proteins Bcl-2, Bcl-xL in the extrinsic apoptotic signaling pathway. It was concluded that APS could not only effectively reduce the MOMP but also inhibit the expression of pro-apoptotic proteins of both the extrinsic and intrinsic pathways and modulate the ratio of Bcl-2 to Bax in mitochondria and finally decrease the high glucose-induced H9C2 cell apoptosis [100] (Figure 3⑦). In addition, the cumulative results show that the pathogenesis of diabetic cardiomyopathy is associated with oxidative stress, apoptosis and proliferation of local cardiac cells. Therefore, inhibiting oxidative stress is believed to be an important strategy for the therapy of diabetic cardiomyopathy. Additionally, previous studies revealed that the neuregulin-1 (NRG1)/ErbB pathway is impaired in diabetic cardiomyopathy cells, which suggested that the NRG1/ErbB pathway may play an important role in diabetic cardiomyopathy [101,102,103]. Moreover, NRG1/ErbB can improve glucose tolerance in healthy and diabetic rodents [103] and regulate the oxidative capacity of myocyte [104,105]. It was confirmed that APS could increase proliferation, inhibit apoptosis and improve antioxidative function including reducing intracellular ROS level, elevating activity of GSH-Px and SOD and lowering the level of MDA and NO by activating the NRG1/ErbB pathway [106] (Figure 3⑧). What is more, substantial reports suggested that the diabetic heart is characterized by reduced glucose metabolism and enhanced fatty acid utilization [107]. Diabetic hearts had elevated rates of fatty acid oxidation, ectopic fat deposition and subsequent lipid peroxidation by PPARα regulatory pathways, leading to lipotoxic cardiomyopathy, which finally resulted in ventricular dysfunction [108,109]. Reduce cardiac fatty acid utilization may improve cardiac performance [110]. Data indicated that the activation of PPARα target genes involved in myocardial fatty acid uptake and oxidation in both db/db diabetic hearts and myosin heavy chain-PPARα hearts was reciprocally repressed by APS administration in db/db diabetic mice [111] (Figure 3⑨).

High glucose-induced inflammation, renal microangiopathy, renal injury and fibrosis are the main characteristics of diabetic nephropathy, which is the leading cause of the end-stage failure of the kidney. NF-κB, an activation of the transcription factor, has been suggested to be a key step in the pathogenesis of diabetic nephropathy. Under physiological conditions, NF-κB is bound with an inhibitory protein of nuclear factor-κB (IκB) to form as an inactive transcription factor in the cytoplasm. After exposure to various stimuli, such as oxidative stress, angiotensins and various cytokines, NF-κB is released rapidly from IκB and then activate the gene expression of several cytokines, chemotactic and matrix proteins involved in inflammation, immunological responses and/or proliferation, which may contribute to the accumulation of renal extracellular matrix and tubulointerstitial fibrosis, resulting in kidney damage [112]. APS was proven to inhibit the expression of inflammatory factors and alleviate diabetic nephropathy by inhibiting the NF-κB signaling pathway in streptozotocin-induced diabetic nephropathy rats [113,114] (Figure 3⑩). In addition, evidence exists that epithelial-mesenchymal transition (EMT) and transforming growth factor (TGF)-β1 play important roles in renal fibrosis. Over-expression of TGF-β1 can promote the expression of its downstream SMAD and then promote the EMT process, thereby promoting renal fibrosis in patients with diabetic nephropathy. It was reported that AS-IV can delay the renal fibrosis process in diabetic KKAy mice by influencing the TGF-β/SMADS signaling pathway and down-regulating TGF-β1, SMAD2/3 and inhibiting the EMT process [115] (Figure 3⑪). Besides, endoplasmic reticulum (ER) stress also plays an important role in the pathogenesis of diabetic nephropathy. The data from a study indicated that AS-IV reduced the ER stress to decrease proteinuria and attenuate diabetes in streptozotocin-induced diabetic rats, which might be an important mechanism in the renoprotective function of AS-IV in the pathogenesis of diabetic nephropathy [116] (Figure 3⑫). What is more, growing evidence suggests that the Wnt/β-catenin pathway and the TGF-β1/Smads pathway are closely linked to the process of cell injury. Overexpression of microRNA-21 can activate the Wnt/β-catenin pathway and TGF-β1/Smads pathway and promote renal injury and fibrosis. It was found that the overexpression of miR-21 activated the β-catenin pathway and the TGF-β1/Smads pathway in the process of podocyte dedifferentiation and mesangial cell activation, which could be abolished by AS-IV treatment. Additionally, AS-IV could also improve renal function and fibrosis in diabetic KK-Ay mice [117] (Figure 3⑬).

Other diabetic complications, including diabetic retinopathy and cognitive dysfunction, also could be effectively alleviated by AR and its bioactive compounds. It was observed that AS-IV could prevent the activation of ERK1/2 phosphorylation and NF-κB and further relieve the retinal ganglion cells dysfunction in db/db mice with diabetic retinopathy, which provided a basis for investigating the clinical efficacy of AS-IV in preventing diabetic retinopathy [116]. In addition, it is known that persistent hyperglycemia can cause brain tissue damage and cognitive impairment in diabetic patients. APS had a hypoglycemic effect on db/db diabetic mice by alleviating the hyperglycemia, tissue impairment and inhibiting cognitive impairment [39].

### 3.3. Effects of AR and Its Main Components on Cancer

AR and its main components are widely used in the treatment of cancer and rehabilitation after operations in patients because of their characteristics of increasing curative effect and reducing the toxicity of chemotherapeutic drugs [11,118]. Substantial experiments have shown that AR and its main active ingredients have pharmacological effects such as inhibiting the proliferation and differentiation of cancer cells, inducing apoptosis of cancer cells, inhibiting invasion and migration of cancer cells, reducing drug resistance and enhancing immune function [15,119]. Growing evidence suggests that the main bioactive compounds responsible for the anti-cancer effects of AR mainly include formononetin, AS-IV and APS. Several mechanisms linking AR and its main components to the regulation of cancer are summarized in Figure 3.

The anti-tumor activity of formononetin has been extensively studied and reported. Formononetin can effectively inhibit various tumors by inhibition of the proliferation, migration and invasion of tumor cells and induction of the tumor cell apoptosis. Genomic studies reveal that PI3K/AKT signaling is one of the most frequently deregulated pathways in several human cancers. This pathway plays a crucial role in cancer cell proliferation, survival, motility and metabolism and therefore could be an attractive therapeutic target. It was found that formononetin could significantly inhibit the PI3K/AKT signaling pathway to induce the apoptosis of cervical cancer HeLa cells and suppress xenograft tumor growth in nude mice, which indicated that formononetin may be used as an anti-cancer drug for cervical cancer in the future [120] (Figure 4①). Another study reported that formononetin also prevented the tumor growth of human breast cancer MCF-7 cells in nude mouse xenografts and caused cell cycle arrest at the G0/G1 phase by inactivating the IGF1/IGF1R-PI3K/Akt pathways, indicating the use of formononetin in the prevention of breast cancer carcinogenesis [121] (Figure 4①). Similarity, two studies found that formononetin could exhibit inhibitory activity against human prostate cancer cells and human non-small cell lung cancer cells through induction of cell cycle arrest and apoptosis in the cancer cells, which demonstrated that formononetin might be a potential chemopreventive drug for therapy lung cancer and prostate cancer [122,123]. Besides, it is well known that ROS takes part in a variety of cellular activities, including survival, proliferation, apoptosis and migration. Low ROS concentration can promote cell survival, while excessive ROS production easily causes DNA damage and induce cell apoptosis [124]. Recently, one study revealed that formononetin could significantly suppress the tumor growth in the multiple myeloma xenograft mouse model without exhibiting any significant adverse effects and exhibit significant anti-cancer effects in multiple myeloma cells that may be primarily mediated through the increased ROS-regulated inhibition of the Janus kinases (JAKs)/signal transducer and transcriptional activators (STATs) signaling cascade [119] (Figure 4②). In addition, Compelling studies have demonstrated that microRNA-21, a putative oncogene, could promote tumor cell proliferation and migration by negatively regulating its target, phosphatase and tensin homolog (PTEN), which functions as a tumor suppressor gene of bladder cancer [125]. It was observed the bladder cancer T24 cells exposed to formononetin displayed obvious morphological changes of apoptosis and lower invasiveness. The expression of microRNA-21 was significantly decreased in T24 cells, followed by an increase of PTEN and down-regulation of a phosphorylated homolog of Akt (p-Akt), which suggest that formononetin exerts an anti-carcinogenic effect on T24 cells via miR-21-mediated regulation of the PTEN/Akt pathway [126] (Figure 4③). What is more, accumulating evidence suggests that chemoresistance is a major obstacle to successful chemotherapy for glioma. Epithelial-mesenchymal transition (EMT), which induces epithelial cells to transform to the mesenchymal phenotype, exerts an important role in regulating the chemoresistance properties of glioma [127]. The histone deacetylase 5 (HDAC5), a member of the class II histone deacetylase family, has been shown to play a critical role in cell proliferation, cell cycle progression and apoptosis, could promote glioma cells proliferation and might provide novel therapeutic targets in the treatment of gliomas [128]. It was found that formononetin may enhance the therapeutic efficacy of doxorubicin in glioma cells by preventing EMT through inhibition of HDAC5 [129] (Figure 4④).

Compelling studies have demonstrated that AS-IV also exhibits inhibitory activity against cancer. For example, AS-IV could inhibit the migration and proliferation of non-small cell lung cancer (NSCLC) cells and caused a noticeable increase in cell death via inhibition of the Akt/GSK-3β/β-catenin signaling axis. The authors, therefore, propose that AS-IV represents a promising novel agent for the treatment of NSCLC [130] (Figure 4⑤). Vav3, a proto-oncogene, has been identified as an important molecule in tumorigenesis, tumor growth and cell migration. Its oncogenic activity is mediated by different downstream pathways, including mitogen-activated protein kinase (MAPK) pathway [131]. The study supports the hypothesis that AS-IV inhibited the viability and invasive potential of MDA-MB-231 breast cancer cells by suppressing Vav3 mediated MAPK signaling pathway and downregulating the expression of matrix metalloproteases (MMP)-2 and -9 [132] (Figure 4⑥). Similarity, AS-IV suppressed the migration and invasion ability of glioma U251 cells via blocking the MAPK/ERK signaling pathway in vitro and in vivo [133]. Besides, accumulating evidence demonstrates that M2-polarized tumor-associated macrophages (TAMs) play a vital role in cancer progression and metastasis, making M2 polarization of TAMs an ever more appealing target for therapeutic intervention. It was observed that AS-IV could reduce the growth, invasion, migration and angiogenesis of lung cancer by blocking the M2 polarization of macrophages partially through the AMPK signaling pathway [134] (Figure 4⑦). In addition, EMT is known as a multistage reprogramming process that promotes metastasis and the initiation and execution of EMT could be triggered by growth factors such as transforming growth factor β1 (TGF-β1) [135]. Evidence exists that AS-IV could inhibit TGF-β1-induced EMT through inhibition of the PI3K/Akt/NF-κB pathway in gastric cancer cells, which suggests that AS-IV might be an effective candidate for the treatment for gastric cancer [136] (Figure 4⑧). Similarity, AS-IV could suppress the levels of inflammatory factors TGF-β1, TNF-α and IL-6 and also decrease the levels of EMT related factors integrin β1, MMP-2 and MMP-9 to inhibit migration and invasion in human lung cancer A549 cells via regulating PKC-α-ERK1/2-NF-κB pathway [137]. What is more, The Fas/FasL signaling pathway is a key modulator of cancer cell apoptosis and reduced Fas/FasL expression conducive to tumor progression. AS-IV also exerts an anti-carcinogenic effect on human osteosarcoma via induction of apoptosis and regulation of caspase-dependent Fas/FasL signaling [138]. Additionally, recent findings suggest that long noncoding RNAs (lncRNAs) have crucial roles in hepatocellular carcinoma (HCC) initiation and progression. Among the lncRNAs, long noncoding RNA activated by TGF-β (lncRNA-ATB) is first identified in HCC [139]. Functionally, lncRNA-ATB promotes EMT and metastasis of HCC cells and also promotes survival of HCC cells via activating IL-11/STAT3 signaling. The data from a study indicated that AS-IV could downregulate lncRNA-ATB expression to repress EMT and migration of HCC cells. Moreover, through downregulating lncRNA-ATB, AS-IV inactivated the IL-11/STAT3 signaling, induced HCC cell apoptosis and decreased HCC cell viability. These data provided a novel molecular basis for the applications of AS-IV in the therapy of HCC [140] (Figure 4⑨).

Accumulating evidence suggests that APS also possesses effective anti-tumor activity. For example, APS can delay the growth of human lung cancer A549 cell line xenograft in BALB/C nude mice in vivo and inhibit the proliferation of human lung cancer cell line A549 and NCI-H358 via the inhibition activity of NF-κB transcription activity [141]. APS also had observable apoptosis-induced effects on human gastric carcinoma MGC-803 cells via arresting the cell cycle in S phase and inducing the intrinsic mitochondrial apoptosis pathway [43]. Besides, Notch protein 1 (Notch 1), which is a transmembrane receptor involved in transcriptional regulation, has been demonstrated to regulate cell proliferation, apoptosis and differentiation in lung carcinoma [142]. The downregulation of Notch1 inhibited cell growth and induced apoptosis in A2780 ovarian cancer cells [143]. It was demonstrated that APS could induce the apoptosis of human HCC cells by decreasing the expression of Notch1 [144] (Figure 4⑩). What is more, APS was also proved to repress proliferation, migration and invasion while induced apoptosis of human osteosarcoma MG63 cells by up-regulating miR-133a and then inactivating JNK pathway [145].

In addition to the single bioactive compounds, the extracts of AR also exhibit inhibitory activity against cancer [146]. For example, the AR water extract could markedly inhibit the proliferation and induce the apoptosis of breast cancer cells, including MCF-7, SK-BR-3 and MDA-MB-231, via inhibiting the PI3K/Akt/mTOR signaling pathway [147] (Figure 4⑪). These findings provide a new insight into the anti-cancer effect of AR extract as a promising agent in breast cancer treatment. Moreover, the antitumor effect of AR water extract was also assessed on the subcutaneous tumors of human colorectal cancer cell line HCT116 grafted into nude mice. The results showed that the AR water extract could inhibit the growth of colorectal cancer in vivo without apparent toxicity and side effect, which suggests that AR is a potential therapeutic drug for colorectal cancer [148].

### 3.4. Effects of AR and Its Main Components on Respiratory Diseases

**Figure 5:**
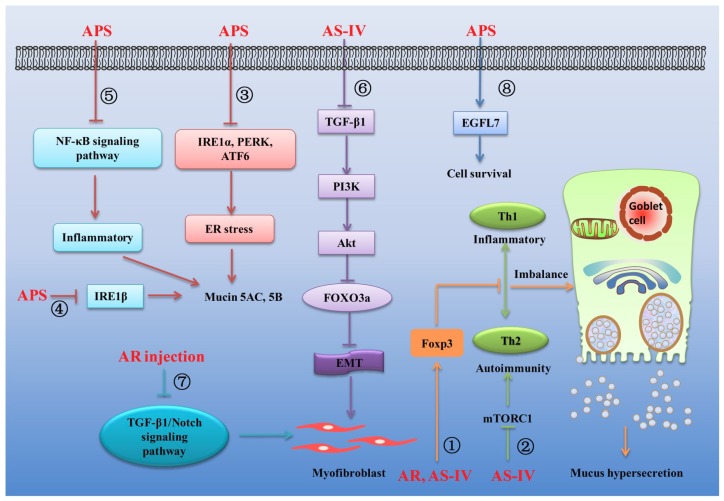
Several mechanisms linking AR and its main components to the treatment of respiratory diseases. Arrows and bar-headed lines represent signaling activation and inhibition, respectively. The different signaling pathways regulated by AR and its components are numbered ① to ⑧.

Respiratory diseases, which mainly include asthma, bronchitis, chronic obstructive pulmonary disease and pulmonary fibrosis, seriously reduce the quality of human life. The main characteristics of respiratory diseases include inflammation, excessive secretion of respiratory mucus and pulmonary fibrosis [149,150]. Substantial evidence exists that AR and its main components exhibit inhibitory activity against a variety of respiratory diseases because of their effective biological functions. The main mechanisms linking the treatment of respiratory diseases by AR and its main components are summarized in Figure 5.

Asthma, a chronic airway inflammatory disease, is associated with a wide range of symptoms, including prolonged inflammation, airway hyperresponsiveness, mucus hypersecretion and airway remodeling [151]. Airway inflammation, one of the distinct characteristics of asthma, is directly linked to a T helper 2 (Th2)-associated disorder due to Th1/Th2 imbalance [152]. The imbalance of Th1 and Th2 cytokines can cause immune dysfunction, which can stimulate goblet cells to secrete excessive mucus in asthma [153]. CD^4+^ CD^25+^ regulatory T cells (Tregs) play a vital role in the regulation of immune function and their roles in asthma pathogenesis are increasingly recognized [154]. The forkhead family transcription factor Foxp3, which is predominantly expressed by CD^4+^CD^25+^ T cells, has been regarded as not only a faithful marker of Tregs but also a critical component for Treg development and function [154]. It was found that AR extract could significantly increase the population of CD^4+^CD^25+^Foxp^3+^ Treg cells, promote Foxp^3+^ mRNA expression, enhance Th2-mediated response and inhibit Th1-mediated response, thereby inhibiting the inflammation in a rat model of asthma, suggesting that the antiasthmatic effects of AR are at least partially associated with CD^4+^CD^25+^Foxp^3+^ Tregs [12] (Figure 5①). Another similar study showed that AS-IV also could attenuate allergic inflammation by regulation Th1/Th2 cytokine and enhancement CD^4+^CD^25+^Foxp^3+^ T cells in ovalbumin-induced asthma [155] (Figure 5①). Besides, previous studies revealed that the mTOR signaling pathway, a downstream signaling pathway of PI3K, is closely related to the proliferation and differentiation of T cells and plays an important role in the pathological process of asthma [156]. Inhibition of mTOR could attenuate key characteristics of allergic asthma, including airway inflammation, airway hyperreactivity and goblet cell metaplasia [157]. It was observed that AS-IV could significantly ameliorate airway inflammation by inhibiting the mTORC1 signaling pathway in an established murine model of asthma [158] (Figure 5②). ER stress, which can induce cell apoptosis by damaging DNA, also play an important role in the development of asthma. PERK (PKR-like ER kinase), IRE1α and -β (inositol-requiring transmembrane kinase/endonucleases) and ATF6 (activating transcription factor 6) are three classes of stress sensors expressed at the ER membrane and are closely involved in the production of ER stress [159]. IRE1β, a subtype of IRE1α, is related to the excessive secretion of mucin 5AC and 5B in the respiratory tract. It was found that APS can inhibit ER stress by inhibiting the expression of IRE1α, PERK and ATF6 [159] (Figure 5③) and decrease the expression of MUC5AC/MUC5B by inhibiting the expression of IRE1β in respiratory tract [160] (Figure 5④). Moreover, APS can inhibit the expression of inflammatory factors by inhibiting NF-κB signaling pathway [161] (Figure 5⑤). Thus, it was concluded that APS could effectively inhibit asthma by inhibiting ER stress and reduce the excessive secretion of respiratory mucus and respiratory inflammation in ovalbumin-induced severe asthma mice [162].

Growing evidence suggests that EMT plays a critical role in the development of pulmonary fibrosis. The cytokine transforming growth factor-β (TGF-β) functions served as an important mediator of fibrogenesis. Plenty of works have identified that TGF-β1 is an important pro-fibrotic factor that has been shown to induce EMT in pulmonary fibrosis [163]. Thus, the anti-EMT pathway or the method of inhibiting of TGF-β1 signaling could provide a novel potential target for the treatment of pulmonary fibrosis. Besides, Forkhead box O transcription factor 3a (FOXO3a) is involved in pulmonary fibrosis. Suppressed FOXO3a activity resulting from hyperphosphorylation of FOXO3a by Akt was found closely linked to the progression of pulmonary fibrosis [164]. It was demonstrated that AS-IV could suppress the TGF-β1/PI3K/Akt pathway to active FOXO3a, thus prevents EMT in bleomycin-induced pulmonary fibrosis [165] (Figure 5⑥). In addition, inhibition of Notch signaling is proved to be a potential therapeutic strategy for pulmonary fibrosis. Another study was designed to investigate the antifibrosis effects and possible mechanism of AR injection on bleomycin-induced pulmonary fibrosis in rats and revealed that AR injection could exert protective effects on bleomycin-induced pulmonary fibrosis via downregulating TGF-β1/Notch1 in lung [166] (Figure 5⑦).

Bronchopulmonary dysplasia (BPD) is defined as the most common form of chronic lung damage in premature infants, which includes barotrauma, volutrauma and oxygen toxicity [167]. It is known that epidermal growth factor-like domain 7 (EGFL7) is secreted by endothelial cells and widely distributed in lung, heart and spleen, plays an important role in angiogenesis. It has been found that EGFL7 gene expression is significantly decreased in neonatal rat lungs following exposure to hyperoxic conditions and has been identified as a potential therapeutic target for lung injury [168]. It was found that APS could exert protective effects in newborn rats with bronchopulmonary dysplasia by upregulating the expression of EGFL7 in lung tissue [169] (Figure 5⑧). In addition, the finding provided evidence that AS-IV could exert protective effects against paraquat-Induced lung Injury in mice by suppressing NF-κB signaling pathway [170]. What is more, another study was conducted to assess the effectiveness and safety of oral AR for preventing frequent episodes of acute respiratory tract infections (ARTIs) in children. The results indicated that the intake of AR alone or in combination with other drugs can effectively enhance the immune function and prevent upper respiratory tract infection in children [171].

### 3.5. Effects of AR and Its Main Components on Nervous System Diseases

**Figure 6:**
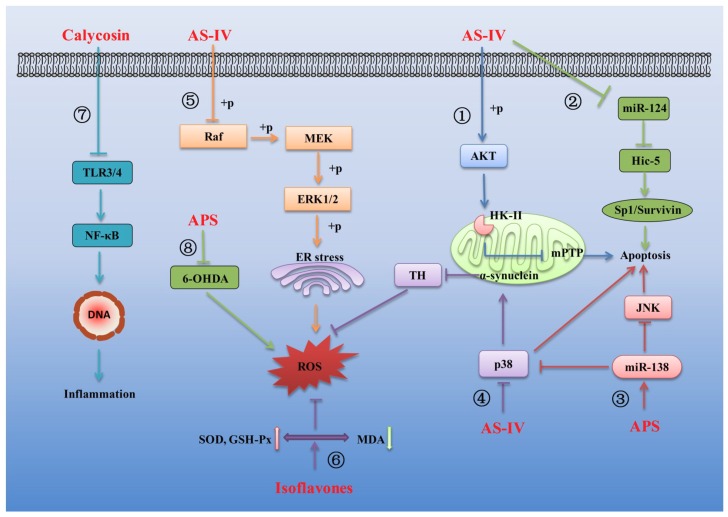
Several mechanisms linking AR and its main components to the treatment of nervous system diseases. Arrows and bar-headed lines represent signaling activation and inhibition, respectively. The different signaling pathways regulated by AR and its components are numbered ① to ⑧.

There is increasing evidence indicating that AR and its main components also exhibit inhibitory activity against a variety of nervous system diseases, mainly including cerebral ischemia injury [13], chemotherapy-induced neuropathy [172] and neurodegenerative diseases [173]. Several mechanisms linking AR and its main components to the treatment of the nervous system diseases are summarized in Figure 6.

It has been proven that mitochondrial dysfunction emerges as a key event linking altered metabolism with neuronal death. The stability of mitochondrial structure and function is essential for neuronal survival during ischemic injury. The binding of hexokinase-II (HK-II) to mitochondria is demonstrated to protect mitochondrial function from ischemic injury [174]. Thus, pharmacological intervention to preserve mitochondrial HK-II is shown to protect mitochondrial function and reduce apoptosis in the ischemic brain [175]. It was found that AS-IV could protect the integrity of mitochondrial function, inhibit the expression of apoptosis-related proteins and protect the ischemic brain tissue damage by activating Akt to promote HK-II binding to mitochondria in ischemic injury mice [176] (Figure 6①). Plenty of studies have identified the functions of microRNAs (miRNAs) in ischemic diseases. Among those identified miRNAs, miR-124 is a specific miRNA in the nervous system and its aberrant expression contributes to the pathological condition related with central nervous system [177]. Hic-5, which is a member of the group III LIM domain protein family and was negatively regulated by miR-124 [178], can activate Sp1/Survivin signaling pathway to regulate the central nervous system injury [179,180]. It was observed that AS-IV could decrease miR-124 expression and then up-regulate Hic-5 expression, thereby activating Sp1/Survivin signaling pathway, exerting anti-apoptotic and anti-cerebral ischemic effects in hypoxia-induced injury PC12 cell [181] (Figure 6②). Another study also demonstrated a protective effect of APS on hypoxia-induced neural stem cell injury by up-regulation of miR-138 and inhibition of the JNK and p38MAPK pathways [182] (Figure 6③). Similarity, AS-IV exerted significant protective effects by decreasing the apoptotic ratio and attenuating ROS overproduction in hydrogen peroxide-exposed human neuronal cells ((SH-SY5Y cells)) by decrease the expression of α-synuclein and to increase the expression of tyrosine hydroxylase (TH) in the cells via the p38MAPK signaling pathway [183] (Figure 6④). Besides, the MAPK pathway plays a vital role as transducers of extracellular stimuli into a series of intracellular phosphorylation cascades, which ultimately leads to cell differentiation, proliferation, survival or death [184,185]. ERK, as one of the major MAPK subfamilies, can be activated by inflammatory cytokines and extracellular stressors [186]. Activation of the Raf-MEK-ERK pathway has been shown to be a key regulator of neuronal apoptosis [187], which makes this pathway an important molecular target of neurodegenerative diseases therapy [188]. Accordingly, a high-throughput comparative proteomic approach based on 2D-nano-LC-MS/MS to investigate the possible mechanism of action involved in the neuroprotective effect of AS-IV against glutamate-induced neurotoxicity in PC12 cells. The data indicated that proteins associated with signal transduction, immune system, signaling molecules and interaction and energy metabolism play important roles in neuroprotective effect of AS-IV and the Raf-MEK-ERK pathway was involved in the neuroprotective effect of AS-IV against glutamate-induced neurotoxicity in PC12 cells [189] (Figure 6⑤).

The isoflavonoids isolated from AR are also receiving much attention due to their various health benefits. Plenty of works have suggested that some of these beneficial effects of isoflavonoids mediated by their antioxidant activity [190]. The neuroprotective roles and direct antioxidant effects of these isoflavonoids were investigated by using a PC12 cell model. The results showed that three of the isoflavonoids, formononetin, ononin and calycosin could prevent the decrease in activity of antioxidant enzymes superoxide dismutase (SOD) and glutathione peroxidase (GSH-Px) and the depletion of GSH in glutamate-damaged PC12 cells, indicating that these compounds could protect PC12 cells from oxidative stress induced by glutamate. These isoflavones could also protect the integrity of membrane structure and inhibit nerve injury by inhibiting the release of lactate dehydrogenase (LDH) in glutamate-induced injury PC12 cell [191] (Figure 6⑥). A brief overview of reports on the potential effects of calycosin on several diseases and the possible mechanisms showed that calycosin possesses the functions of antioxidant and neuroprotective role by inhibiting the phosphorylation of ERK1/2 in the downstream of the MAPK signaling pathway and could effectively inhibit the production of ROS/MDA and promote the production of SOD/GSH-Px [55]. In addition, the previous studies implied that AR extract can be a potential nerve growth-promoting factor, being beneficial for the growth of peripheral nerve axons [192]. A recent study also supported that AR extract can modulate local inflammatory conditions, enhance nerve regeneration and potentially increase recovery of a severe peripheral nerve injury in a rat sciatic nerve transection model [193]. The protective properties of AR extract against oxaliplatin-induced neurotoxicity were also investigated. AR extract showed significant antioxidant and protective effects against oxaliplatin-induced lipid peroxidation and DNA oxidation in oxaliplatin-induced nerve injury SH-SY5Y cells [172].

Parkinson’s disease (PD), an incurable progressive disease, is characterized by shaking, rigidity, slowness of movement and difficulty with walking [194]. Some antioxidants and anti-inflammatory drugs can relieve experimental Parkinson’s symptoms [195,196]. It is well known that NF-κB is a family of transcription factors, which have an effect on regulation of inflammatory response [65,66,67,68]. TLRs, a class of pattern recognition receptors, can regulate NF-κB and are involved in various inflammatory responses [197,198]. The expression levels of TLR2 and TLR4 significantly increased in the blood and brain of PD patients [199]. Besides, the MAPK pathway also plays a crucial role in the inflammatory response [200]. It was found that calycosin could attenuate 1-methyl-4-phenyl-1,2,3,6-tetrahydropyridine (MPTP)-induced PD by suppressing the activation of the TLR/NF-κB and MAPK pathways, indicating the potential drug of calycosin against PD [201] (Figure 6⑦). In addition, APS also have a very good therapeutic effect in the treatment of PD. It was confirmed that APS could attenuate MPTP-induced motor dysfunction, increase the proportion of TH-positive cells, reverse MPTP-induced mitochondrial structural damage and reduce MPTP-induced high levels of ROS and increase MPTP-induced decrease in mitochondrial membrane potential. Moreover, APS also decreased the bax/bcl2 ratio and cytochrome-c and caspase-3 protein expression in substantia nigra in a mouse PD model [202]. Similarity, APS was shown to have the protective effect against 6-hydroxydopamine (an oxidative metabolite of dopamine) induced PD, which is likely due to the alleviation of oxidative stress and regulation of the apoptosis pathway and cholinergic system [203] (Figure 6⑧).

### 3.6. Other Pharmacological Activities

In addition to cardiovascular diseases, diabetes mellitus, cancer, respiratory diseases and nervous system diseases, AR and its bioactive compounds are also reported to have other various therapeutic uses. For example, AS-IV could protect against the progression of renal fibrosis by inhibiting inflammation via the TLR4/NF-κB signaling pathway [204]. APS possessed high potential in wound healing, which was associated with inhibiting inflammation, accelerating cell cycle and promoting the secretion of repair factors [205]. AR extract could attenuate inflammation and oxidative stress in intestinal epithelial cells via NF-κB activation and Nrf2 response [206]. AR extract could also reduce intestinal mucosal damage and promote tissue repair by inhibiting the expression of inflammatory cytokine in LPS-induced intestinal mucosal injury mice [18]. What is more, it was proven that AR extract could also reduce the production of melanin and inhibit melanogenesis through activating of the ERK signaling pathway in melanoma B16F10 cells [207].

Conclusively, as an indispensable herbal drug widely used in clinical practice, AR has played a crucial role in healthcare. An increasing number of extracts and active compounds have been isolated and immunomodulation, antioxidant, anti-inflammation and antitumor activities, among others, were found. The effective treatment of cardiovascular diseases, diabetes mellitus, cancer, respiratory diseases and nervous system diseases by AR were also reported and verified by in vivo and in vitro experiments. With the increasing popular studies on AR and the development of models in the biological evaluation, more and more pharmacological activities of AR and its bioactive compounds will be clarified in the future.

## 4. Pharmacokinetic Studies

### 4.1. Pharmacokinetic Studies on AR Extracts

The pharmacokinetics of the AR water extract in rats have been reported in our previous study [20]. Eight compounds, including four parents formononetin, calycosin-7-β-glucoside (CG), ononin, AS-IV and four metabolites calycosin-3′-glucuronide (C-3′-G), calycosin-7-β-glucoside-3′-glucuronide (CG-3′-G), formononetin-7-glucuronide (F-7-G) and daidzein-7-glucuronide (D-7-G), could be detected after single oral administration of AR water extract at doses of 4 g/kg and 15 g/kg in male rats [20]. The results showed that isoflavonoids and their metabolites are the major type of constituents absorbed in plasma and the plasma concentrations of the four metabolites were much higher than their parents. Interestingly, daidzein, an aglycone compound, was not found in the AR water extract but its metabolite D-7-G could be detected in the plasma, which could be speculated that daidzein could be generated from formononetin, ononin, calycosin and its glycoside through demethylation, dehydroxylation and deglycosylation in the gastrointestinal tract and then was glucuronidated to D-7-G in the enterocytes. Moreover, the plasma concentration of calycosin, a major constituent in AR, was too low to be detected. The one reason may be that the rate of glucuronidation of calycosin was very fast in the enterocytes and few calycosin entered the systemic circulation; another may be that some calycosin in the enterocytes could be excreted into the intestinal tract by transporters and were circulated between the enterocytes and the gastrointestinal tract. An earlier similar study reported the absorption and metabolism of AR water extract using computational chemistry prediction method, Caco-2 cell monolayer model experiment, improved rat everted gut sac experiment and healthy human volunteer experiment [23]. According to in silico computation result, 26 compounds of AR could be regarded as oral available compounds, including 12 flavonoids. In the in vitro and in vivo experiments, 21 compounds were tentatively identified, which involved the parent compounds and their metabolites. The flavonoids in AR, including isoflavones, pterocarpans and isoflavans, could be absorbed and metabolized by the intestine and the major metabolites were glucuronides. In addition, the absorbed components and their pharmacokinetic profile after oral administration of AR water extract were investigated on cyclophosphamide-induced immunosuppression in Balb/c mice [21]. As a result, 51 compounds in AR extract and 31 prototype compounds with nine metabolites were detected in mice plasma. It was also observed that flavonoids and saponins as the two main kinds of constituents were detected in plasma after the mice were administrated AR orally. Among the absorbed constituents, 11 flavonoids and 12 triterpenoid saponins accounted for almost three-quarters of the 31 compounds. In addition, The pharmacokinetic studies on AR ethanol extract were also reported [22]. Six main bioactive components, including CG, ononin, calycosin, formononetin, AS-IV and astragaloside II in rat plasma were detected after oral administration of the 95% ethanol extraction from AR.

Furthermore, the pharmacokinetic behaviors of the bioactive compounds after oral administration of AR extracts were also analyzed. As shown in Table 2, the eight major compounds, including formononetin, CG, ononin, AS-IV and their metabolites C-3′-G, CG-3′-G, F-7-G and D-7-G, achieved their maximum plasma concentrations within 1 h, demonstrating rapid absorption from the gastrointestinal tract in rats [20]. Among these compounds, C_max_ of metabolite C-3′-G was highest and followed by metabolite F-7-G. While their parent compounds CG and ononin had lowest C_max,_ which suggested that CG and ononin could rapidly transform to their glucuronides through hydrolyzation and glucuronidation. Moreover, the eight detected compounds had relative slow elimination [20]. In another study, the pharmacokinetic studies of five bioactive compounds, including CG, ononin, calycosin, formononetin, AS-IV, were analyzed after oral administration of AR water extract in mice [21]. In the four isoflavonoids, formononetin showed relatively slow absorption and elimination with t_max_ at 2 h and t_1/2_ at 3.99 h (Table 2). Meanwhile, the double-peak phenomenon as a common phenomenon in the absorption of AR flavonoids was found. It was speculated that this phenomenon possibly resulting from enterohepatic circulation, transformation of different compounds, double-site absorption and intestinal efflux. In addition, AS-IV showed relatively more absorption and slower excretion reflected by the data of AUC_0–t_ 695.37 ± 178.57 μg/L·h, C_max_ 128.95 μg/L and t_1/2_ 3.48 ± 1.15 h (Table 2). The pharmacokinetic studies of the bioactive compounds after oral administration of AR 95% ethanol extract in rats were also reported [22]. It was observed that among the four isoflavones (CG, ononin, calycosin and formononetin) detected in the plasma and calycosin was absorbed and eliminated the most rapidly with t_max_ at 1.0 h and t_1/2_ at 3.880 h (Table 2). Formononetin was absorbed and eliminated the most slowly among four isoflavones with t_max_ at 2.5 h and t_1/2_ at 1.207 h (Table 2). What’s more, the values of C_max_ and AUC of calycosin and formononetin in plasma were higher than ononin and CG, although the contents of calycosin and formononetin in AR were markedly lower than ononin and CG (Table 2). It was speculated that this phenomenon might be a part of ononin and CG which was at first hydrolyzed by enzymatic action to corresponding formononetin and calycosin in vivo and attributed to the biotransformation of flavonoid glycosides to aglucones by intestinal bacteria and enzymes in vivo, giving rise to a great increase of formononetin and calycosin in plasma.

**Figure 7:**
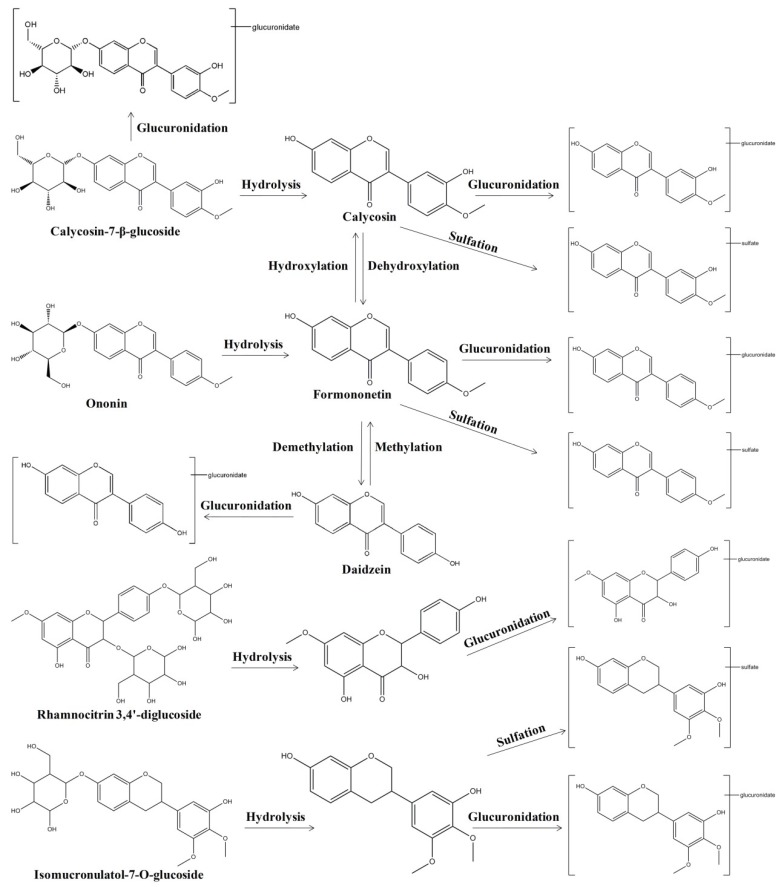
Proposed pathways of metabolism and biotransformation of the compounds after administration of AR extracts in vivo.

Collectively, these studies demonstrated that isoflavonoids and saponins and their metabolites are the major type of constituents absorbed in plasma after oral administration of AR extracts. The metabolism and biotransformation were extensive after administration of AR extracts in vivo (Figure 7). 

### 4.2. Pharmacokinetic Behaviors of Bioactive Compounds from AR

#### 4.2.1. Drug-Metabolizing Enzymes and Drug Transporters

The bioavailability barrier (BB), which mainly consists of drug-metabolizing enzymes (DMEs) and efflux transporters (ETs), abundantly distributed in the liver and intestine of the human body [24,208]. The coupled metabolic activities of DMEs and ETs control the absorption, distribution, metabolism and excretion of drugs being taken up by the human body. That is, these coupling processes represent a significant barrier to the oral bioavailability of drugs [24,25,26]. DMEs, including phase I and phase II metabolizing enzymes, play key roles in the metabolism, elimination and/or detoxification of xenobiotics or exogenous compounds introduced into the body [209,210]. Cytochromes P450 (CYPs), abundantly found in the liver, gastrointestinal tract, lung and kidney, are the main Phase I enzymes [209]. Phase II enzymes, which are mostly transferases, include sulfotransferase (SULT), glutathioneS-transferase (GST), uridine 5′-diphospho (UDP)-glucuronosyltransferase (UGT), N-acetyltransferases (NATs) and various methyltransferases [209,210,211]. The human ETs, which mainly include P-glycoprotein (P-gp), multidrug resistance-associated protein 2 (MRP2) and breast cancer resistance protein (BCRP), are abundantly expressed in many tissues such as the intestine, liver, kidney and brain and also play key roles in drug absorption, distribution and excretion [208,212,213]. There is increasing evidence indicating that the major metabolic pathway for flavonoids is phase II conjugation. Two superfamilies of phase II enzymes, UGTs and SULTs, catalyze the formation of hydrophilic phase II conjugates for flavonoids [24,214]. Plenty of works have also identified that the ETs are involved in the cellular excretion of flavonoids and their phase II conjugates [24,214]. On the one hand, once metabolized to hydrophilic metabolites, these hydrophilic conjugates have to be transported out of cells by the transporters that are localized in the intestine [24,214]. On the other hand, flavonoids and their conjugates, which are transported to the liver, may be subjected to additional metabolism or excretion via the bile [24,214,215]. Taken together, the coupling of ETs and conjugating enzymes are expected to have a significant impact on the bioavailability and apparent half-life of the flavonoids.

#### 4.2.2. Formononetin

Formononetin, a methoxylated isoflavone, is one of the vital bioactive compounds of AR and possesses various pharmacological activities [8]. The pharmacokinetic parameters of formononetin and daidzin were determined in rats following intravenous and oral administration of formononetin (Table 3). It was observed that formononetin was extensively converted to its metabolites daidzin, daidzin and formononetin conjugates (glucuronides and/or sulfates). The plasma concentrations of conjugates of formononetin and daidzin, which were generated after the enzymatic hydrolysis with UGTs, were markedly higher than the parent compound formononetin and daidzin [216]. In another study, the parallel artificial membrane permeability assay permeability, protein binding, blood uptake characteristics, pharmacokinetics and metabolism of formononetin were analyzed in rats [217]. The permeability of formononetin was found to be high. Formononetin was rapidly absorbed into the systemic circulation, which may be due to the high permeability and lipophilic nature. The oral bioavailability of formononetin is very low (near 3%), which may be due to extensive first-pass metabolism by phase I oxidative metabolism and phase II glucuronidation and/or sulfation in the intestine and liver. The AUC of formononetin and daidzin conjugates (glucuronides and/or sulfates) were higher than formononetin and daidzin, indicating that formononetin and daidzin conjugates are the main existent form in rat plasma after oral and intravenous administration. The reentry peaks of formononetin, daidzin and their conjugates were present in the plasma, likely due to enterohepatic recirculation, after oral as well as intravenous administration. The red blood cells uptake of formononetin was independent of the initial rat blood concentrations and time. The plasma protein binding of formononetin was observed to be high (more than 93%). In addition, the pharmacokinetics, bioavailability and in vitro absorption of formononetin after oral and intravenous administration in rats were investigated [218]. It was found that formononetin was moderately bioavailable in rats with an absolute bioavailability of 21.8% and was absorbed in all gastrointestinal segments with varied permeability. In the Caco-2 model, formononetin showed moderate absorption via passive diffusion. What is more, studies about the transport and metabolism of formononetin in vivo and in vitro were also reported [215,219,220,221,222]. The results showed that glucuronide was the main metabolite in intact Caco-2 cell model. Coupling of the conjugating enzymes and ETs determines the rate and direction of conjugate excretion. The excretion of formononetin glucuronide is mediated by the multidrug resistance-related proteins (MRPs) and organic anion transporters (OATs) that are located at the basolateral and apical membranes of the Caco-2 cells. 

#### 4.2.3. Calycosin-7-β-d-Glucoside (CG)

CG, a marker used routinely to monitor the quality of AR, exhibits several pharmacological activities [223]. The pharmacokinetic parameters after the administration of CG in rats were displayed in Table 3. In one study, the factors influencing CG absorption and disposition were determined [223]. After oral administration of CG, CG and its metabolites rapidly appeared in the plasma sample. CG, C-3′-G was found to be the main form circulating in plasma, suggesting that deglycosylation of CG to calycosin was an important step before entry into systemic circulation. After intraperitoneal administration of CG, CG rapidly entered the systemic circulation and became the main circulating component in the plasma, thereby limiting hydrolysis in the liver. Using a rat intestinal perfusion model, CG showed good absorption and CG transport from the lumen to enterocytes was mediated by sodium-dependent glucose transporter 1 (SGLT-1). CG was likely to be hydrolyzed in enterocytes to its aglycone calycosin by broad-specific β-glucuronides (BSβG) and glucocerebrosidase or rapidly metabolized. Then, calycosin was also rapidly and extensively metabolized by UGTs to 3′-glucuronide in the enterocytes and liver. Moreover, the metabolites were also transported into the lumen by BCRP and MRP2. What is more, the pharmacokinetic characteristics of CG were determined after oral administration of CG in rats [224]. As for the low cumulative urinary excretion of CG after oral administration, little CG was excreted into urine as the archetype, suggesting that CG may be metabolized by intestinal bacteria. The low concentration detected in the rat plasma, which indicated that little CG could be absorbed into the plasma from the rat gastrointestinal tract. The hepatic first-pass effect, as well as the UGT-mediated metabolism, could account for the low detection of CG in plasma and urine.

#### 4.2.4. Ononin

Ononin, the glycoside form of formononetin, is also one of the major isoflavonoids in AR with biological activities [218]. The pharmacokinetic parameters after the administration of ononin in rats were displayed in Table 3. The pharmacokinetics, bioavailability and in vitro absorption of ononin were investigated after oral or intravenous administration to rats [218]. After an oral administration, ononin showed a double-peak phenomenon in the plasma concentration profile and the first peak was observed at around 30 min, indicating a rapid absorption of ononin. It has also been demonstrated that ononin could be biotransformed into formononetin via colonic microbiota-mediated deglycosylation [231]. Moreover, ononin may be deglycosylated by the lactase-phlorizin hydrolase, an enzyme found on the brush border of the mammalian small intestine in the small intestine [232]. Ononin had a relatively low bioavailability of 7.3%. Since ononin was rapidly biotransformed in the gut to generate formononetin and further metabolized [231]. Besides, after an oral administration, ononin had a small absorption in the small intestine and most parts were absorbed in the large intestine. In all segments of intestines, the permeability of ononin was poor, which is proposed as an alternative contributor for its poor bioavailability. In addition, bidirectional transport assay was performed to investigate whether efflux transporters were involved in the absorption of ononin [218]. In was observed that ononin was a substrate of MRP2 but not P-gp and its low bioavailability might be partially attributed to MRP2-mediated efflux during absorption.

#### 4.2.5. Astragaloside IV (AS-IV)

As the most abundant saponin and a characteristic compound of AR, AS-IV is documented as the chemical marker for the quality control of AR [233]. The pharmacokinetic behavior of pure AS-IV ingested by rats and dogs have been widely studied and clearly elucidated (Table 3). In an early study, the pharmacokinetics and tissue distribution of AS-IV in both rats and dogs were examined [226,234]. After intravenous administration in rats, AS-IV showed moderate to fast elimination and was slowly cleared via hepatic clearance. AS-IV showed an extensive distribution into multiple tissues followed by a rapid elimination from most of the tissues. The highest concentration of AS-IV was detected in the lung and liver. However, limited distribution to the brain indicates that AS-IV may have difficulty penetrating the blood-brain barrier. In addition, only about 50% of the parent AS-IV was recovered in both urine and feces, suggesting that near 50% of AS-IV may undergo metabolism in vivo. After intravenous administration in dogs, AS-IV also showed moderate to fast elimination and was slowly cleared via hepatic clearance. In another study in beagle dogs [230], it was found that the absolute bioavailability of AS-IV in Beagle dogs was fairly low (7.4%), which would restrict its application in oral administration. It was also confirmed that AS-IV has low gastrointestinal tract absorption and bioavailability in rats in another study [225]. Moreover, the protein binding rate of AS-IV was about 90%, which means that that AS-IV bound extensively to plasma proteins and that most of it formed associative patterns in dog plasma [230]. The authors claimed that this should be taken into account when AS-IV is considered for clinical use together with other high protein binding drugs. In addition, two studies reported the absorption of AS-IV in the perfused rat intestinal model, transport and uptake in Caco-2 cell monolayers and in vivo bioavailability in rats after an oral dose [227,235]. It was found that the transport of AS-IV was predominantly via a passive route. AS-IV having a low fraction dose absorbed in humans mainly due to its poor intestinal permeability, high molecular weight, low lipophilicity as well as its paracellular transport may directly result in the low permeability through its passive transport. Besides, AS-IV was proved not to be a substrate of P-gp that its poor absorption is not due to the action of this efflux protein. What is more, in recent research, the authors profiled the metabolites of AS-IV in rat plasma, bile, urine and feces samples after oral administration [236]. A total of 22 metabolites, including phases I and II metabolites, was detected and tentatively identified. The major metabolic pathways of AS-IV in rats were hydrolysis, glucuronidation, sulfation and dehydrogenation. Additionally, AS-IV is metabolized into the aglycon or deglycosylated in the gastrointestinal tract and absorbed only partially. In another recent study, the authors investigated the enterohepatic circulation of AS-IV and evaluated the impact of activity of intestinal microbiota on the deposition of AS-IV in rats [228]. After oral or intravenous administration, AS-IV was metabolized by intestinal bacteria to form brachyoside B (Bra B), cyclogaleginoside B (Cyc B), cycloastragenol (CA), iso-cycloastragenol (iso-CA) and a dehydrogenated metabolite of CA (CA-2H). AS-IV and its two metabolites CA and iso-CA circulated in the blood. The plasma distribution of AS-IV was significantly affected by bile duct drainage when AS-IV was administrated through the duodenum. After rats were pretreated with antibiotics, the metabolism of AS-IV in intestinal microbiota was markedly inhibited. Moreover, variations in intestinal microbiota may change the disposition of AS-IV. A similar study also confirmed that the bacteria play a crucial role in the metabolism and biotransformation of AS-IV in vivo [237].

## 5. AR as Personalized Medicine

As concluded above, AR possesses various biological functions, which has played an indispensable role in healthcare throughout Chinese history for thousands of years. However, so far, the relationship between the dose and efficacy of AR has still not been clearly clarified, which is reflected in the curative effects of AR vary greatly between different individuals. According to the historical literature and modern clinical reports, the dose of AR administration range from 0.1 g to 300 g [238], which far exceeds the doses of up to 30 g recorded in the latest Chinese Pharmacopoeia. Among 504 patients with syndrome of blood stasis, only 62 patients displayed markedly attenuated disease after oral AR administration at the dose of less than 30 g and most of the patients were insensitive to the curative effects of AR [239]. Among another 166 patients with similar syndrome and orally administrated with AR at the dose of more than 30 g, only 39 patients were cured, which corresponded to a cure rate of 7.5% [239]. In addition, among 274 patients with malignant tumor, AR administration at different doses could irregularly alleviate the adverse reactions caused by chemotherapy drugs [240,241]. These clinical statistics clearly showed that there are significant differences in the reactions to AR efficacies among different patients with the similar syndrome. Therefore, the personalized use of AR represents a novel strategy that is necessary for obtaining AR with enhanced efficacy and precise treatment.

The BB, which mainly consists of DMEs and ETs, abundantly distributed in the liver and intestine of the human body [24,208]. On the one hand, the BB acts as a critical xenobiotic detoxification system, which plays a crucial role in protecting the human body from the possible toxicity of external chemicals. On the other hand, the BB controls the absorption, distribution, metabolism and excretion of drugs being taken up by the human body. That is, the BB regulates the pharmacokinetic behavior of drugs in vivo, thereby determining the concentration of drugs [24,25,26]. Thus, there is a close link between the BB network and drug efficacy. Diverse polymorphisms of DMEs and ETs in individuals can markedly influence the pattern or pathway coupling in the BB network, which can notably produce different pharmacokinetic behavior of drugs and ultimately results in variations in the efficacy of drugs in different individuals. Therefore, the BB network could be used as a key determinant for clinical implementing personalized medicine.

From the results of the pharmacokinetic studies on AR concluded above, it has been well-recognized that the coupling of UGTs and ETs plays a crucial role in regulating the bioavailability of the bioactive compounds from AR. Thus, it can be speculated that the diverse polymorphisms of UGTs and ETs in patients with the similar syndrome could notably produce differences in the reactions to the AR efficacies, which would lead to blindness in treatment and could easily result in ineffective treatment. Transcriptome studies showed that the genetic polymorphisms of UGTs extensively exist in different individuals, which directly regulates the efficacy and toxicity of drugs in different individuals [242,243,244]. For example, patients carrying UGT1A1*28 allele(s) are at an increased risk of medium or high dose of irinotecan-induced severe diarrhea [242], those patients with wild-type in UGT family genes have lower rates of toxicity associated with irinotecan treatment than those with certain mutated allele [243]. In addition, genetic polymorphism in UGT1A4 and UGT2B7 may play a modest role in lamotrigine clearance changes during pregnancy [244]. Furthermore, it has been confirmed that a large number of polymorphisms at the UGT1A and UGT2B genes have been shown to modulate UGT gene promoter activity or enzymatic activity. Thus, UGT polymorphisms that reduce the capacity to glucuronidate carcinogens and other types of cancer-promoting molecules (e.g., sex hormones) are associated with an increased risk of developing cancers [245]. Moreover, the UGT1A4*3 polymorphism is an independent risk factor for being a poor absorber of posaconazole oral suspension in patients with hematological malignancies [246]. The genetic polymorphisms of ETs, which can markedly alter the pharmacokinetic behaviors of drugs, are also closely related to the toxicity and efficacy of clinical drugs. For example, the P-gp (ABCB1, MDR1) 1199G > A polymorphism may impact effective antipsychotics concentration in target cells via mediating the agents transport and distribution [247]. Significant associations between sorafenib exposure and the studied polymorphisms were observed for ABCB1 3435C > T SNP in the patients during the treatment with sorafenib of hepatocellular carcinoma [248]. Rheumatoid arthritis-patients carrying wild-type alleles of MDR1 might benefit from P-gp inhibition or administration of glucocorticoid analogs that are non-P-gp substrates [249]. Besides, the body disposition of lumefantrine is also notably influenced by MRP2/ABCC2 genotype among patients with uncomplicated plasmodium falciparum malaria [250]. The ABCC2 polymorphism 1249G > A increases the ATPase activity of MRP2, leading to greater efflux of sorafenib [251]. ABCC2 genetic polymorphisms also strongly influence inter-individual variation of telmisartan pharmacokinetics in the renal transplant recipients [252]. Growing evidence suggests that genetic polymorphisms of ABCG2 are also closely related to inter-individual variations in therapeutic performance. BCRP (ABCG2) 34 G > A and ABCG2 1143 C > T polymorphisms were significantly associated with the lowest sorafenib plasma levels of the patients during the treatment with sorafenib of hepatocellular carcinoma [248]. The common single nucleotide polymorphism c.421C > A, p.Q141K reduces cell surface expression of ABCG2 protein, resulting in the lower efflux of substrates. Consequently, a higher plasma concentration of substrate is observed in patients carrying an ABCG2 c.421C > A allele [253].

Collectively, the clinical use of AR as a personalized medicine is necessary to achieve a better and more accurate therapeutic efficacy. The polymorphisms in UGTs and ETs are important determinants of inter-individual differences in the toxicities and response to many drugs and also plays a crucial role in regulating the pharmacokinetic behavior of AR in vivo. Thus, the genetic polymorphisms of the UGTs and ETs in different individuals can be applied to characterize and optimize personalized therapies of AR in clinics in the future.

## 6. Conclusions and Future Prospects

As an effective and safe herbal drug widely used to treat various diseases, AR has played an indispensable role in healthcare in China and other countries. It is worth mentioning that AR is a popular herb frequently coadministered with many other herbal or chemical drugs for efficacy improvement in clinics. According to the historical literature and modern clinical reports, AR has been used as an essential ingredient in over 200 Chinese herb prescriptions for treating various complicated diseases. However, the potential risks of herb-herb or herb-drug interactions intensively exist during the combination therapy in clinics, which could easily cause an undesirable variation in the plasma concentrations of coadministered drugs, thereby resulting in treatment failure or toxicologically unsafe consequences [254,255,256]. Therefore, the potential herb-herb or herb-drug interactions arising from the coadministration of AR and other herbal or chemical drugs must be evaluated. Further studies need to be conducted to confirm the safe combination therapy of AR.

Modern pharmacological studies and clinical practices provide more and more evidence for the effective functions of AR. According to the results from various validated in vivo and in vitro studies, AR has been proved to possesses multiple biological functions, such as immunomodulation, antioxidant, anti-inflammation and antitumor properties and thus widely ingested for the treatment of cardiovascular diseases, diabetes mellitus, cancer, respiratory diseases, nervous system diseases and other diseases. In the last decade, the underlying molecular mechanisms involved in the therapeutic effects of AR have been widely studied and reported. But further investigation is needed because of the various and complicated compounds contained in AR, which leads to the diversity and complexity of the molecular targets intervened by AR.

The phytochemical studies indicate that AR mainly contains isoflavonoids, triterpene saponins, polysaccharides and some other trace elements. To date, more than 200 compounds have been isolated and identified from AR and various biological activities of the compounds have been confirmed. Among them, isoflavonoids, saponins and polysaccharides are the three main types of beneficial compounds responsible for the pharmacological activities and therapeutic efficacy of AR. Further studies need to be conducted to isolate and identify the new compounds from AR to get a more comprehensive understanding of AR.

The pharmacokinetic characteristics of AR and its bioactive compounds have also been widely performed and clearly elucidated. It is well-recognized that the metabolism and biotransformation of the bioactive compounds were extensive after ingestion of AR. The isoflavonoids and saponins and their metabolites are the major type of constituents absorbed in plasma. The coupling of UGTs and ETs in vivo plays a crucial role in regulating the pharmacokinetic characteristics and determining the bioavailability of the bioactive compounds from AR. Thus, the varied polymorphisms of UGTs and ETs in different individuals are likely to be the critical factors contributing to the vast dosage differences in AR ingestion in individuals. In future clinical practice, the genetic polymorphisms of the UGTs and ETs in different individuals can be applied to characterize and optimize personalized therapies of AR to achieve better and more accurate therapeutic efficacy.

## Figures and Tables

**Figure 4:**
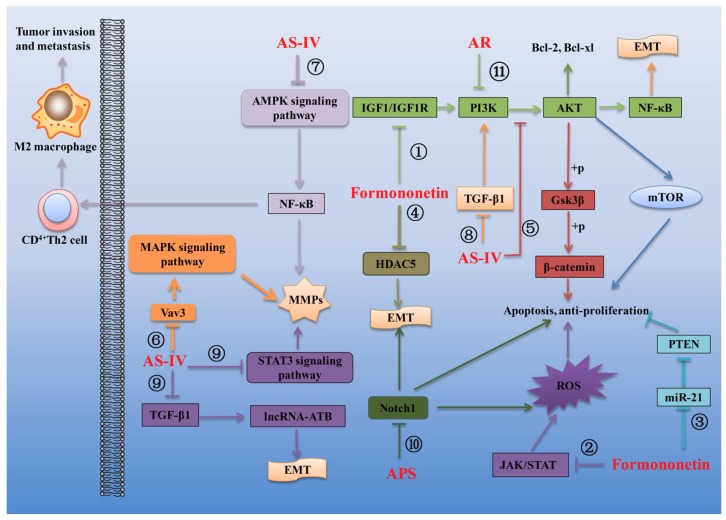
Several mechanisms linking AR and its main components to the treatment of cancer. Arrows and bar-headed lines represent signaling activation and inhibition, respectively. The different signaling pathways regulated by AR and its components are numbered ① to ⑪.

**Table 1:** The main parts of compounds isolated from AR.

NO.	Name	Categories	References
1	Astragaloside I–VIII	*Astragalus* triterpene saponins	[27,29]
2	Acetylastragaloside		[29]
3	Isoastragaloside I–IV		[27,29,30]
4	Acetylastragaloside I		[31]
5	Astramembrannin II		[29]
6	Cycloastragenol		[29]
7	Cyclosieversigenis		[29]
8	Soyasaponin I, II		[29,30]
9	Soyasapogenol B		[29]
10	Lupeol		[29]
11	Malonylastragaloside I		[29]
12	Agroastragaloside I–IV		[30]
13	Monghocoside I, II		[30]
14	Atramembrannin I,II		[30]
15	Asernestioside A, B, C		[30]
16	Astrasieversianin II,X		[30,32]
17	Astrojanoside		[30]
18	Astrojanoside A		[33]
19	Azukisaponin II, V		[30]
20	Brachyoside A, B, C		[30,33]
21	β-daucosterol		[30]
22	β-sitosterol		[30]
23	Cloversaponin IV		[30]
24	Cycloaraloside A		[30]
25	Cyclocanthoside A, B, E, G		[30,34]
26	Cyclocephaloside I, II		[30]
27	Cyclodissectoside		[30]
28	Cyclounifolioside B		[30]
29	Dehydroazukisaponin V		[30]
30	Calycosin-7-*O*-β-d-glucoside		[30]
31	Calycosin-3-*O*-β-d-glucoside		[35]
32	Formononetin-7-*O*-β-d-glucoside		[30]
33	Hareftoside A, B, C, D, E		[30]
34	Isoliquiritigenin		[30]
35	Macrophyllosaponin B		[30]
36	Melilotus-saponinO2		[30]
37	Mongholicoside A, B		[30]
38	Oleifoloside B		[30]
39	Quercetin-3-glucoside		[30]
40	Rhamnocitrin-3-glucoside		[30]
41	Trojanoside A, B, H		[30]
42	Wistariasaponin B2, D		[30]
43	2′-hydroxy-3′,4′-dimethoxyisoflavone-7-*O*-β-d-glucopyranoside		[30]
44	2′-hydroxy-3,4′-dimethoxyisoflavan-7-*O*-β-d-glucoside		[35]
45	3′,4′-dimethoxyisoflavone-7-*O*-β-d-glucoside		[30]
46	3′-methoxy-5′-hydroxy-isoflavone-7-*O*-β-d-glucoside		[30]
47	3-*O*-β-d-xylopyranosyl-6,25-di-*O*-β-d-glucopyranosyl-3β,6α,16β,24(S),25-pentahydroxycycloartane		[30]
48	3-*O*-β-d-xylopyraosyl-24S-cycloart-3β,6α,16β,24,25-pentaol-25-*O*-β-d-glucopyranoside		[30]
49	3-*O*-[α-l-rhamnopyranosyl-(1→2)-β-d-xylopyranosyl-(1→2)-β-d-glucuronopyranosyl]-3β,21β,22α,24,29-pentahydroxyolean-12-ene		[30]
50	3-*O*-β-d-glucuronopyranosyl-soyasapogenin B		[30]
51	6,3′-dihydroxy-2′,4′-dimethoxyisoflavean-6-*O*-β-d-glucopyranoside		[30]
52	7,3′-dihydroxyl-6,4′-dimethoxyisoflavon-7-*O*-β-d-glucopyranoside		[30]
53	7,2′-dihydroxy-3′,4′-dimethoxyisoflavan-7-*O*-β-d-glucoside		[30]
54	(6αR,1lαR)-3-hydroxy-9,10-dimethoxypterocarpan-3-*O*-β-d-glucoside		[29,30]
55	(3R)-2′-hydroxy-3′,4′-dimethoxyisoflavan-7-*O*-β-d-glucoside		[30,36]
56	(6αR,11αR)-9,10-dimethoxypterocarpan-3-*O*-β-d-glucoside		[30]
57	(3R,4R)-3-(2-hydroxy-3,4-dimethoxyphenyl)chroman-4,7-diol-7-*O*-β-d-glucopyranoside		[30]
58	7-methylquercetin-3-*O*-α-l-rhamnopyranosyl-(1→2)-[6-*O*-(3-hydroxy-3-methylglutaryl)-β-d-galactopyranoside]		[37]
59	kaempferol 3-*O*-α-l-rhamnopyranosyl-(1→2)-[6-*O*-(3-hydroxy-3-methylglutaryl)-β-d-galactopyranoside]		[37]
60	7-methylkaempferol 3-*O*-α-l-rhamnopyranosyl-(1→2)-β-d-galactopyranoside		[37]
61	7-methylkaempferol-3-*O*-α-l-rhamnopyranosyl-(1→2)-[6-*O*-(3-hydroxy-3-methylglutaryl)-β-d-galactopyranoside]		[37]
62	7-methylque-rcetin 3-*O*-β-d-galactopyranoside		[37]
63	20(R),24(S)-epoxy-9β,19-cyclolanostane-3β,6α,16β,25-tetrol 3-*O*-α-l-rhamnopyranosyl-(1→4)-β-d-glucopyranoside		[33,38]
64	20(R),24(S)-epoxy-9β,19-cyclolanostane-3β,6α,16β,25-tetrol 3-*O*-α-l-rhamnopyranosyl-(1→2)-β-d-glucopyranoside		[33,38]
65	20(R),24(S)-epoxy-9β,19-cyclolanostane-3β,6α,16β,25-tetrol 3-*O*-β-d-glucopyranoside		[38]
66	20(R), 25-epoxy-9β, 19-cyclolanostane-3β, 6α, 16β, 24(S)-tetrol (24-*O*-acetyl)-3-*O*-α-l-rhamnopyranosyl-(1→2)-(6′-*O*-acetyl)-β-d-glucopyranoside		[38]
67	Calycosin-7-*O*-β-d-glucoside-6″-*O*-acetate	*Astragalus* flavonoids	[30]
68	Calycosin-7-*O*-β-d-glucoside-6″-*O*-malonate		[29]
69	Calycosin		[29]
70	Ononin		[29]
71	Formononetin-7-*O*-β-d-glucoside-6″-*O*-malonate		[29]
72	Formononetin		[29]
73	Dimethoxy-dihydrogen-isoflavones		[30]
74	Astrapterocarpan-glucoside-6″-*O*-malonate		[29]
75	Astraisoflavan-7-*O*-β-d-glucoside-6″-*O*-malonate		[35]
76	Sulfuretin		[27]
77	Pendulone		[27]
78	Isoliquiritigenin		[27]
79	Rutin		[36]
80	Cascara citrin		[30]
81	(3R)-8,2′-dihydroxy-7,4′-dimethoxyisoflavan		[36]
82	Dimethoxy isoflavones		[30]
83	Isoliquiritigenin,dimethoxy ispflavan		[30]
84	Isorhamnetin		[30]
85	Kaempferol		[30]
86	Kumatakehin		[30]
87	l-3-hydroxv-9-methoxypterocarpan		[30]
88	Pterocarpans		[30]
89	Quercetin		[30]
90	Rhamnocitrin		[30]
91	Sphondin		[30]
92	Kaempferol		[37]
93	2′-hydroxy-3′,4′-dimethoxyisoflavone-7-*O*-β-d-glucopyranoside		[30]
94	2′-hydroxy-3′,4′,7-trimethoxyisoflavone		[30]
95	2′,3′,7-trihydroxy-4′-methoxyisoflavone		[30]
96	2′,4′-dihydroxv-5,6-dlmethoxvlsoflavane		[30]
97	4,2′,4′-trihydroxy chalcone		[30]
98	5,7,4′-trihydroxyisoflavone		[30]
99	8,2′-dihydroxy-4′,7-dimethoxyisoflavone		[30]
100	(3R)-7,2′-dihydroxy-3′,4′-dimethoxyisoflavan		[30]
101	(3R)-2′,3′-dihydroxy-4′,7-dimethoxyisoflavone		[30]
102	3,9,10-trimethoxypterocarpan,(6αR,1lαR)-10-hydroxy-3,9-dimethoxypterocarpan		[30]
103	9,10-dimethoxypterocarpan-7-*O*-β-d-glucopyranoside		[30]
104	3-hydroxy-9,10-dimethoxypterocarpan		[35]
105	APS A,B, C, D	*Astragalus* polysaccharides	[29]
106	AERP1 (Molecular weight: 2.01 × 10^6^ Da)		[39]
107	AERP2 (Molecular weight: 2.11 × 10^3^ Da)		[39]
108	APS (Glc, Ara, Gal and Rha)		[40]
109	APS (Glc)		[40]
110	APS (Glc, Molecular weight: 2.1 × 10^4^ Da)		[40]
111	APS-I (Ara: Xyl: Glc in the ratio of 0.54: 1: 18.14, Molecular weight: 4.8×10^6^ Da)		[40]
112	APS-II (Ara: Xyl: Glc in the ratio of 0.23: 1: 29.39, Molecular weight: 8.7×10^3^ Da)		[40]
113	APS (Glc, Molecular weight: 3.6×10^4^ Da)		[40]
114	APS-I (Glc: Gal: Ara in the ratio of 1.75: 1.63: 1, Molecular weight: 3.6×10^4^ Da)		[40]
115	APS-II (Glc, Molecular weight: 1.2 × 10^4^ Da)		[40]
116	APS-III (Glc, Molecular weight: 3.5 × 10^4^ Da)		[40]
117	APS (Man, Gal, Fru, Fuc and Xyl)		[40]
118	Astragalan (Glc, Molecular weight: 1.5 × 10^4^ Da)		[40]
119	APS (GIc: Gal: Ara in the ratio of 1.75: 1.63: 1, Molecular weight: 3.6×10^4^ Da)		[40]
120	APS (Glc, Molecular weight: 3.6 × 10^4^ Da)		[40]
121	APS (Rha: Glc: Gal: Ara in the ratio of 1.19: 72.01: 5.85: 20.95, Molecular weight: 1.1×10^4^ Da)		[40]
122	AMon-S (Ara: Gal: GalA: GlcA in the ratio of 18: 18: 1: 1, Molecular weight: 7.6 × 10^4^ Da)		[40]
123	F-8 (Rha: Rib: Fuc: Ara: Xyl: Man: Gal: GIc in the ratio of 2: 2: 1: 2: 6: 2: 3: 100, Molecular weight: 2.2 × 10^4^ Da)		[40]
124	F-9 (Fuc: Xyl: GIc in the ratio of 1: 2: 100, Molecular weight: 2.2 × 10^4^ Da)		[40]
125	APS (Rha: Xyl: GIc: Gal: Man: Fru in the ratio of 4.9: 4.7: 8.3: 122.2: 2.2: 3.1)		[40]
126	APSID3(Ara: Rha: Gal: Glc in the ratio of 2: 2: 5: 6, Molecular weight: 5.8 × 10^5^ Da)		[40]
127	APS-I (Ara: GIc in the ratio of 1: 3.45, Molecular weight: 1.7 × 10^6^ Da)		[40]
128	APS-II (Rha: Ara: GIc in the ratio of 1: 6.25: 17.86, Molecular weight: 1.2 × 10^6^ Da)		[40]
129	APS (Ara: Man: GIc: Gal in the ratio of 0.10: 1.26: 1: 0.01)		[40]
130	APS (Molecular weight: 6.9 × 10^4^ Da)		[40]
131	APS (Rha: Xyl: GIc: Gal in the ratio of 1: 4: 5: 1.5, Molecular weight: 3.0 × 10^5^ Da)		[40]
132	RAP (Rha: Ara: Glc: Gal: GalA in a molar ratio of 0.03: 1.00:0.27: 0.36: 0.30, Molecular weight: 1.334 × 10^6^ Da)		[41]
133	APS (Rha: Xyl: Gle: Gal: Man: Fru in a molar ratio of 4.9: 4.7: 8.3: 122.2: 2.2: 3.1, Molecular weight: 3500~ 1.58 × 10^6^ Da)		[42]
134	APS4 (Rha: Ara: Xyl: Man: Gal in a molar ratio of 12.1: 0.3: 0.6: 1.0: 1.0: 1.7, Molecular weight: 1.61 × 10^6^ Da)		[43]
135	Arabino-3,6-galactan		[44]
136	alcohol-soluble polysaccharide (ASP) (Ara: Gal: Glu: Man in a molar ratio of 1.00: 0.98: 3.01: 1.52, Molecular weight: 2100 Da)		[5]

**Table 2:** Pharmacokinetic parameters of detected compounds following oral administration of AR extracts.

Administration	Species	Dose	Detected Compounds	Pharmacokinetic Parameters						
				C_max_ (ng/mL)	T_max_ (min)	AUC_(0-t)_ (min·ng/mL)	AUC_(0-∞)_ (min·ng/mL)	MRT (h)	T_1/2_ (min)	CL/F (L·h^−1^·kg^−1^)
HQ aqueous extract, p.o., single treatment [20]	Rat	4 g/kg	CG	2.67 ± 1.17	28.33 ± 4.08	355.48 ± 96.91	399.66 ± 138.44	ND	177.24 ± 73.98	ND
			Ononin	3.97 ± 0.83	22.50 ± 6.12	425.26 ± 59.89	451.147 ± 65.53	ND	163.22 ± 34.44	ND
			Formononetin	4.24 ± 1.62	18.33 ± 6.06	341.31 ± 108.69	385.78 ± 114.41	ND	213.09 ± 55.57	ND
			AS-IV	8.40 ± 5.64	45.00 ± 25.10	2777.4 ± 1220.25	3321.99 ± 1032.04	ND	291.83 ± 125.58	ND
			C-3′-G	1841.99 ± 391.56	33.33 ± 13.66	370,570.07 ± 118,683.13	423,856.39 ± 128,163.25	ND	232.59 ± 112.86	ND
			F-7-G	141.23 ± 54.67	33.33 ± 9.83	27,808.62 ± 5918.16	30,076.63 ± 6376.79	ND	182.50 ± 56.30	ND
			CG-3′-G	88.94 ± 40.61	45.00 ± 9.49	10,550.06 ± 5895.49	10,580.22 ± 5897.22	ND	70.56 ± 9.35	ND
			D-7-G	19.65 ± 10.15	35.83 ± 14.29	2145.60 ± 574.75	2232.42 ± 614.78	ND	140.30 ± 25.94	ND
HQ aqueous extract, p.o., single treatment [20]	Rat	16 g/kg	CG	17.27 ± 10.19	25.00 ± 5.48	1256.42 ± 555.23	1405.16 ± 505.53	ND	313.14 ± 188.96	ND
			Ononin	9.52 ± 4.20	24.17 ± 6.65	865.61 ± 349.91	858.61 ± 352.27	ND	68.71 ± 15.26	ND
			Formononetin	26.01 ± 9.75	10.83 ± 2.04	2534.14 ± 942.59	3053.52 ± 1243.25	ND	280.86 ± 115.56	ND
			AS-IV	26.63 ± 13.54	21.67 ± 1.67	3237.24 ± 993.96	4116.24 ± 1078.26	ND	319.91 ± 58.10	ND
			C-3′-G	3301.60 ± 1113.35	25.00 ± 10.95	746,605.22 ± 105,842.50	860,702.31 ± 269,532.48	ND	185.70 ± 176.90	ND
			F-7-G	282.21 ± 131.2	48.33 ± 24.63	64,267.55 ± 20,197.31	66,083.40 ± 20,987.58	ND	125.20 ± 26.86	ND
			CG-3′-G	97.08 ± 38.95	42.50 ± 6.12	16,864.73 ± 6773.30	16,945.86 ± 6736.94	ND	95.84 ± 57.09	ND
			D-7-G	50.75 ± 32.36	50.00 ± 20.49	9847.21 ± 7727.72	10,978.84 ± 7912.12	ND	255.71 ± 124.07	ND
HQ aqueous extract, p.o., single treatment [21]	Mice	6 g/kg	CG	33.41	90.00	6489.60 ± 2228.40	7630.20 ± 2418.60	ND	156.60 ± 40.20	ND
			Ononin	51.38	90.00	13,670.40 ± 3581.40	14,354.40 ± 2842.80	ND	117.60 ± 20.40	ND
			Calycosin	32.98	90.00	8152.80 ± 2484.6	9292.20 ± 2261.40	ND	132.60 ± 55.20	ND
			Formononetin	47.93	120.00	14,002.80 ± 2869.80	15,385.20 ± 2740.80	ND	239.40 ± 61.80	ND
			AS-IV	128.95	90.00	41,722.20 ± 10,714.20	43,349.40 ± 11,791.20	ND	208.80 ± 69.00	ND
HQ 95 % ethanol extract, p.o., single treatment [22]	Rat	11.36 g/kg	CG	0.205	90.00	ND	56.22	4.273	162.18	97,008.817
			Ononin	0.074	90.00	ND	760.38	4.104	55.86	572,786.673
			Calycosin	0.700	60.00	ND	122.64	3.422	232.80	44,469.181
			Formononetin	0.097	150.00	ND	14.94	4.369	72.42	365,548.079
			AS-IV	0.5879	90.00	ND	107.256	5.210	76.518	50,848.964
			Astragaloside II	0.016	60.00	ND	4.20	4.223	41.76	1,301,557.754

p.o., oral administration; ND, not determined; C-3′-G, calycosin-3′-glucuronide; CG, calycosin-7-β-glucoside; CG-3′-G, CG-3′-glucuronide;F-7-G,formononetin-7-glucuronide; D-7-G,daidzein-7-glucuronide; AS-IV, astragaloside IV.

**Table 3:** Pharmacokinetic parameters of detected compounds following oral/intravenous/intraperitoneal administration of bioactive compounds from AR.

Administration	Species	Dose	Detected compounds	Pharmacokinetic parameters								
				C_max_ (ng/mL)	T_max_ (h)	AUC_(0-t)_ (h·ng/mL)	AUC_(0-∞)_ (h·ng/mL)	MRT (min)	T_1/2_ (h)	CL (L·h^-1^·kg^-1^)	V_d_ (L/kg)	V_ss_ (L/kg)
Formononetin, p.o. [217]	Rat	50 mg/kg	Formononetin	16.67 ± 6.60	1.00 ± 0.00	251.44 ± 63.67	278.27 ± 66.68	ND	ND	ND	ND	NA
			Daidzin	8.99 ± 3.29	ND	145.58 ± 34.70	ND	ND	ND	ND	ND	NA
			Daidzin + Daidzin conjugates	301.33 ± 149.92	ND	5032.25 ± 530.22	ND	ND	ND	ND	ND	NA
			Formononetin conjugates	311.97 ± 97.36	ND	5231.46 ± 1219.91	ND	ND	ND	ND	ND	NA
			Daidzin conjugates	292.70 ± 146.20	ND	4886.67 ± 1498.49	ND	ND	ND	ND	ND	NA
			Formononetin + Formononetin conjugates	327.67 ± 100.72	3.42 ± 2.74	5482.90 ± 1276.11	6509.66 ± 720.47	ND	ND	ND	ND	NA
Formononetin, i.v. [217]	Rat	10 mg/kg	Formononetin	4548.50 ± 321.73	NA	1949.10 ± 295.09	1975.88 ± 317.15	ND	1.95 ± 0.48	5.13 ± 0.82	14.16 ± 1.26	NA
			Daidzin	434 ± 19.80	NA	279.72 ± 52.03	ND	ND	ND	ND	ND	NA
			Daidzin + Daidzin conjugates	494 ± 5.66	NA	1663.89 ± 5.72	ND	ND	ND	ND	ND	NA
			Formononetin conjugates	962.00 ± 311.13	NA	3003.64 ± 321.79	ND	ND	ND	ND	ND	NA
			Daidzin conjugates	308.20 ± 95.32	NA	1356.60 ± 42.89	ND	ND	ND	ND	ND	NA
			Formononetin + Formononetin conjugates	5180 ± 367.70	NA	4976.60 ± 648.18	5098.57 ± 765.53	ND	5.09 ± 1.61	ND	14.23 ± 2.41	NA
Formononetin, i.v. [216]	Rat	10 mg/kg	Formononetin	3207	NA	1683.86	1773.40	ND	10.34	5.63	84.14	NA
			Formononetin + Formononetin conjugates	3676	NA	4317.62	4595.71	ND	7.18	2.17	22.54	NA
			Daidzin	305.73	NA	243.68	263.86	ND	2.53	ND	ND	NA
			Daidzin + Daidzin conjugates	347.73	NA	1625.02	1773.89	ND	6.85	ND	ND	NA
Formononetin, p.o. [218]	Rat	20 mg/kg	Formononetin	81.04 ± 9.63	0.5 ± 0.0	191.38 ± 12.39	203.26 ± 12.93	ND	2.10 ± 0.28	ND	NA	NA
Formononetin, i.v. [218]	Rat	4 mg/kg	Formononetin	349.48 ± 34.63	NA	174.99 ± 23.90	184.97 ± 24.71	ND	2.23 ± 0.6	4.3 ± 0.8	13.9 ± 1.0	ND
CG, i.v. [223]	Rat	0.5 mg/kg	CG	446.1 ± 84.6	NA	89.33 ± 17.00	89.83 ± 17.00	ND	0.38 ± 0.14	ND	ND	ND
			CG glucuronide	5.41 ± 1.22	0.57 ± 0.30	10.00 ± 5.17	12.83 ± 5.33	ND	0.95 ± 0.33	ND	ND	ND
			C-3′-G	10.11 ± 3.17	0.32 ± 0.09	34.00 ± 23.83	54.83 ± 50.55	ND	4.11 ± 3.41	ND	ND	ND
CG, p.o. [223]	Rat	10 mg/kg	CG	7.46 ± 3.76	0.28 ± 0.11	5.33 ± 1.83	5.50 ± 2.67	ND	1.52 ± 0.46	ND	ND	NA
			CG glucuronide	51.29 ± 15.7	1.00 ± 0.39	106.33 ± 55.17	106.67 ± 55.50	ND	1.03 ± 0.24	ND	ND	NA
			C-3′-G	1189.66 ± 346.95	0.96 ± 0.43	1574.50 ± 346.00	1852.67 ± 569.67	ND	2.86 ± 1.21	ND	ND	NA
CG, i.p. [223]	Rat	10 mg/kg	CG	965.24 ± 133.53	0.25 ± 0.06	1199.67 ± 373.83	1210.67 ± 368.00	ND	1.29 ± 0.30	ND	ND	NA
			CG glucuronide	322.24 ± 125.53	1.10 ± 0.42	1681.33 ± 642.67	2422.50 ± 1118.33	ND	6.65 ± 4.85	ND	ND	NA
			C-3′-G	221.62 ± 86.60	1.10 ± 0.42	1151.83 ± 443.33	1651.83 ± 765.00	ND	6.57 ± 4.82	ND	ND	NA
CG, p.o. [224]	Rat	120 mg/kg	CG	1870.00 ± 360.00	0.67 ± 0.08	1595.33 ± 472.33	1679.67 ± 509.00	ND	0.19 ± 0.02 (Ka); 0.26 ± 0.02 (Ke)	71.40 ± 16.20	ND	NA
Ononin, p.o. [218]	Rat	20 mg/kg	Ononin	(1) 2.11 ± 11.23 (2) 4.63 ± 3.62	(1) 0.5 ± 0.0 (2) 4.0 ± 0.0	68.95 ± 16.83	74.59 ± 18.55	ND	1.82 ± 0.56	ND	ND	NA
			Formononetin	(1) 44 ± 2.71 (2) 0.52 ± 3.35	(1) 1.0 ± 0.0 (2) 5.7 ± 0.0	85.09 ± 13.17	ND	ND	NA	ND	ND	NA
Ononin, i.v. [218]	Rat	4 mg/kg	Ononin	491.61 ± 44.42	NA	189.17 ± 23.29	199.62 ± 25.87	ND	1.92 ± 0.8	6.6 ± 0.9	18.4 ± 1.5	ND
AS-IV, p.o. [225]	Rat	20 mg/kg	AS-IV	92.4 ± 14.2	1.0 ± 0.5	419.50 ± 126.65	420.41 ± 129.01	ND	t_1/2α_ = 0.89 ± 0.37; t_1/2β_ = 22.02 ± 8.26	0.045 ± 0.013	0.19 ± 0.15	NA
AS-IV, i.v. [226]	Rat (male)	0.75 mg/kg	AS-IV	3790	NA	ND	4816.67	122	1.64	0.3	0.39	0.63
AS-IV, i.v. [226]	Rat (female)	0.75 mg/kg	AS-IV	5180	NA	ND	2550	47.4	0.57	0.3	0.14	0.23
AS-IV, i.v. [226]	Rat (male)	1.5 mg/kg	AS-IV	6980	NA	ND	6700	95.2	1.12	0.42	0.43	0.71
AS-IV, i.v. [226]	Rat (female)	1.5 mg/kg	AS-IV	4800	NA	ND	4966.67	90.1	1.12	0.18	0.16	0.23
AS-IV, i.v. [226]	Rat (male)	3 mg/kg	AS-IV	7790	NA	ND	8666.67	98.7	1.20	0.36	0.38	0.57
AS-IV, i.v. [226]	Rat (female)	3 mg/kg	AS-IV	7240	NA	ND	9183.33	149	2.48	0.18	0.21	0.41
AS-IV, i.v. [226]	Dog (male)	0.25 mg/kg	AS-IV	1110 ± 280	NA	ND	1218.33 ± 516.67	71.5 ± 7.0	0.87 ± 0.14	0.24 ± 0.06	0.23 ± 0.05	0.32 ± 0.13
AS-IV, i.v. [226]	Dog (female)	0.25 mg/kg	AS-IV	1130 ± 40	NA	ND	1096.67 ± 300.00	82.8 ± 29	1.05 ± 0.37	0.24 ± 0.06	0.22 ± 0.01	0.36 ± 0.04
AS-IV, i.v. [226]	Dog (male)	0.5 mg/kg	AS-IV	4390 ± 2600	NA	ND	2600.00 ± 121.67	75.5 ± 6.9	1.00 ± 0.14	0.24 ± 0.06	0.14 ± 0.07	0.32 ± 0.09
AS-IV, i.v. [226]	Dog (female)	0.5 mg/kg	AS-IV	3480 ± 1600	NA	ND	2883.33 ± 766.67	78.0 ± 8.5	1.12 ± 0.13	0.24 ± 0.06	0.16 ± 0.07	0.31 ± 0.06
AS-IV, i.v. [226]	Dog (male)	1 mg/kg	AS-IV	7920 ± 3700	NA	ND	6033.33 ± 1316.67	83.0 ± 23	1.15 ± 0.35	0.24 ± 0.06	0.14 ± 0.07	0.28 ± 0.08
AS-IV, i.v. [226]	Dog (female)	1 mg/kg	AS-IV	8860 ± 2500	NA	ND	5916.67 ± 1366.67	64.8 ± 16	0.84 ± 0.22	0.18 ± 0.06	0.12 ± 0.04	0.23 ± 0.04
AS-IV, i.v. [227]	Rat	2.5 mg/kg	AS-IV	ND	NA	ND	5990	ND	3.37	0.42	0.77	ND
AS-IV, p.o. [228]	Rat	20 mg/kg	AS-IV	374	0.43	ND	1062	ND	t_1/2α_ = 0.30; t_1/2β_ = 4.65;	0.43	0.56	NA
AS-IV, p.o. [229]	Rat	10 mg/kg	AS-IV	27.08 ± 16.17	2.00 ± 63	85.56 ± 43.17	ND	ND	1.71 ± 1.16	ND	ND	NA
			Cycloastragenol	3.48 ± 2.34	9.67 ± 0.82	13.15 ± 8,78	ND	ND	ND	ND	ND	NA
			Iso-cycloastragenol	7.02 ± 3.68	8.00 ± 0.00	38.23 ± 17.22	ND	ND	4.77 ± 3.26	ND	ND	NA
AS-IV, i.v. [229]	Rat	1.5 mg/kg	AS-IV	7865.40 ± 570.67	NA	7917.21 ± 1038.52	ND	ND	1.29 ± 0.41	ND	ND	ND
			Cycloastragenol	1.21 ± 0.22	8.33 ± 0.82	2.73 ± 0.79	ND	ND	ND	ND	ND	ND
			Iso-cycloastragenol	2.98 ± 0.86	8.00 ± 0.00	15.80 ± 2.60	ND	ND	3.05 ± 1.06	ND	ND	ND
AS-IV, i.v. [230]	Dog	0.5 mg/kg	AS-IV	ND	NA	1998.22	2104	222.61	2.953	0.24	ND	0.74
AS-IV, i.v. [230]	Dog	1 mg/kg	AS-IV	ND	NA	4380	4604.67	232.48	3.28	0.24	ND	0.61
AS-IV, i.v. [230]	Dog	2 mg/kg	AS-IV	ND	NA	11706.67	12075.17	241.80	4.03	0.18	ND	0.61
AS-IV, p.o. [230]	Dog	10 mg/kg	AS-IV	ND	NA	3368.33	3400.83	261.14	3.83	0.6	ND	2.61

p.o., oral administration; i.v., intravenous administration; i.p., intraperitoneal administration; C-3′-G, calycosin-3′-glucuronide; CG, calycosin-7-β-glucoside; CG-3′-G, CG-3′-glucuronide; F-7-G, formononetin-7-glucuronide; D-7-G, daidzein-7-glucuronide; AS-IV, astragaloside IV. NA, not applicable; ND, not determined.

## Abbreviations

AR	*Astragali* radix
BB	Bioavailability barrier
DMEs	Drug-metabolizing enzymes
ETs	Efflux transporters
HPGPC	High-performance gel-permeation chromatography
GC	Chromatography
NF-κB	Nuclear factor-kappa B
TLR4	Toll-like receptor 4
PGC-1α	Peroxisome proliferator-activated receptor-γ coactivator 1 α
NFATc3	Nuclear factor of activated T cell scytoplasmic 3
CaMKII	Calmodulin II kinase
SERCA2a	Sarcoplasmic reticulum Ca2+-ATPase
PPARα	Proliferator-activatedαreceptor
α-SMA	α-smooth muscle actin
ECM	Extracellular matrix
Col-1	Colgen I
TRPM	Transient receptor potential channel
TRPC6	Transient receptor potential channel 6
ROS	Reactive oxygen species
CT-1	Cardiotrophin-1
HO-1	Heme oxygenase-1
NQO1	NAD(P)H dehydrogenase (quinone) 1
Nrf2	Nuclear factor-erythroid 2-related factor 2
LPS	Lipopolysaccharide
tBHP	Tert-butyl hydroperoxide
SOD	Superoxide dismutase
CHD	Coronary heart disease
NOD	Non-obese diabetic
gal-1	Galectin-1
Glut4	Glucose transporter 4
AMPK	AMP-activated protein kinase
PKB	Protein kinase B
PTP1B	Protein tyrosine phosphatase 1B
ER	Endoplasmic reticulum
ATF6	Transcription activator 6
MOMP	Mitochondrial extracorporeal membrane permeability
PPAR	Peroxisome proliferator-activated receptor
MCP-1	Monocyte chemotactic protein-1
ICAM-1	Intercellular adhesion molecule-1
iNOS	Inducible nitric oxide synthase
EMT	Epithelial-mesenchymal transition
STZ	Streptozotocin
IGF1	Insulin-like growth factor 1
PI3K	Phosphatidylinositol3-kinase
Akt	Protein kinase B
GRP78	Glucose-regulated protein 78
FasL	Fas ligand
HDAC5	Histone deacetylase 5
CAV-1	Caveolin-1
TGF-β	Transforming growth factor-β
ECM	Extracellular matrix
LDHA	Lactate dehydrogenase A
TIGAR	TP53-induced glycolysis and apoptosis regulator
MAPK	Mitogen-activated protein kinase
ERK	Extracellular signalregulated kinase
TNF	Tumor necrosis factor
IFN	Interferon
KMT2D	Histone-lysine N-methyltransferase 2Dlysine (K) -specific methyltransferase 2D
CREBBP	CREB binding protein
Foxo3a	Forkhead box O transcription factor 3a
BIP	Binding immunoglobulin protein
CHOP	Chomologous protein
EGFL7	Epidermal growth factor-like domain 7
HK-II	Hexokinase-II
mPTP	Mitochondrial permeability transition pore
PD	Parkinson’s disease
TH	Tyrosine hydroxylase
MPTP	1-methyl-4-phenyl-1
6-OHDA	6-hydroxydopamine
IRS1	Insulin receptor substrate 1
APS	Astragalus Polysaccharides
AS-IV	Astragaloside IV
ISO	Isoproterenol
CG	Calycosin-7-β-glucoside
C-3′-G	Calycosin-3′-glucuronide
CG-3′-G	CG-3′-glucuronide
F-7-G	Formononetin-7-glucuronide
D-7-G	Daidzein-7-glucuronide
CYPs	Cytochromes P450
SULT	sulfotransferase
GST	GlutathioneS-transferase
UGT	Uridine 5′-diphospho (UDP)-glucuronosyltransferase
NATs	N-acetyltransferases
P-gp	P-glycoprotein
MRP2	Multidrug resistance-associated protein 2
BCRP	Breast cancer resistance protein
MRPs	Multidrug resistance-related proteins
OATs	Organic anion transporters
SGLT-1	Sodium-dependent glucose transporter 1
BSβG	Broad-specific β-glucuronides
LPH	Lactase phlorizin hydrolase
Bra B	Brachyoside B
Cyc B	Cyclogaleginoside B
CA	Cycloastragenol
iso-CA	Iso-cycloastragenol
CA-2H	Dehydrogenated metabolite of CA
AUC	Area under the curve
T_1/2_	Elimination half-life
CL	Clearance
V_d_	Apparent volume of distribution
MRT	Mean residence time
